# Early-life development of the microbiome and resistome in antibiotic-naïve dairy calves

**DOI:** 10.1128/spectrum.02510-25

**Published:** 2026-04-30

**Authors:** Maggie M. Murphy, Lee J. Pinnell, Enrique Doster, Cory A. Wolfe, Lance A. Baker, Vinicius S. Machado, Paul S. Morley

**Affiliations:** 1 VERO-Veterinary Education, Research, and Outreach Program, Texas A&M University, Canyon, Texas, USA; 2 Department of Agricultural Sciences, West Texas A&M University 14741, Canyon, Texas, USA; 3 Department of Veterinary Sciences, Texas Tech University, Lubbock, Texas, USA; University of Arkansas Fayetteville animal science, Fayetteville, Arkansas, USA

**Keywords:** cattle, feces, microbial ecology, antimicrobial resistance, organic

## Abstract

This study aimed to characterize early-life changes in the fecal microbiome and resistome of calves. Fecal samples were collected from 49 Holstein heifers born and raised at a large organic dairy in Texas without antimicrobial drug exposures. Samples were collected from five age groups: early pre-weaning at 2–3 days old (Pre 1), late pre-weaning at 5 weeks old (Pre 2), prior to weaning at 12–13 weeks old (Pre 3), post-weaning in group hutches at 12–13 weeks old (Post 1), and later post-weaning at 13–14 weeks old (Post 2). Fecal samples were analyzed using 16S rRNA gene sequencing to characterize microbial communities and target-enriched shotgun sequencing to characterize antimicrobial resistance genes in the resistome. Richness of microbial communities increased as calves aged through the Pre 1, 2, and 3 samplings, before plateauing in the Post 1 and 2 groups. Diversity also increased in the Pre 1 and 2 groups, remaining similar thereafter. In contrast, resistome richness and diversity decreased during early life and then stabilized at around 5 weeks of age (Pre 2). Changes in microbial community structures were dramatic during the first 12 weeks, largely due to a significant decrease in the relative abundance (RA) of Pseudomonadota (Proteobacteria) and an increase in the RA of Bacillota (Firmicutes) and Bacteroidota. The resistome changed with an increased RA of tetracycline resistance genes, while drug and biocide resistance genes decreased. The apparent stabilization of microbial community features after 12 weeks of age may reflect a period when gut microbiome structure begins to establish greater stability.

**IMPORTANCE:**

Early-life development of the gut microbiome can have lasting effects on animal health, immune maturation, and productivity. Using 16S rRNA gene sequencing together with target-enriched metagenomic sequencing, we provide an in-depth characterization of the fecal microbiome and resistome of antibiotic-naïve dairy calves during early life. We demonstrate that microbiome diversity increased with age while resistome diversity decreased, revealing distinct temporal trajectories and suggesting ecological succession as a potential driver of resistance gene dynamics independent of antimicrobial drug exposure. Major resistome features appeared to stabilize earlier than overall microbiome structure, highlighting critical windows in early development when resistance gene composition may be most dynamic. These findings establish an important baseline for interpreting microbiome-resistome interactions and for evaluating how management practices and antimicrobial exposures may influence calf health and antimicrobial resistance ecology in dairy production systems.

## INTRODUCTION

The microbiome of young animals plays a critical role in immunity and metabolism and is therefore of particular importance to livestock producers seeking to optimize animal health and performance (1). Microbial communities undergo rapid changes early in life and exert lasting effects on host physiology and disease risk. A growing body of literature demonstrates that perturbations of the microbiome during early childhood, often described as “the first 1,000 days” from birth to 2 years of age, can predispose individuals to obesity, metabolic disorders, autoimmune conditions, and infectious diseases (2). Antimicrobial exposure is commonly cited as a major contributor to early-life gut microbial dysbiosis in children (3). Dietary factors may also play an important role; young intensively managed calves that are fed grain in early life have been suggested to experience gut barrier dysfunction and microbiome alterations at a very young age (4). Together, these observations indicate that disruptions in the normal establishment of the gut microbiome during early life may increase the risk of adverse health outcomes later in life.

Research has documented that microbial colonization occurs rapidly in young animals, and the community composition changes quickly (5, 6). In calves, previous studies have shown that by 9–13 weeks of age, the gut microbiome resembles that of more mature calves, although previous studies were limited by relatively shallow sequencing depth (7). Importantly, antimicrobial resistance genes (ARGs) within the microbial community (the resistome) are also established early in calves and are influenced by the dams’ colostrum and their diet (6). Understanding factors that can influence the establishment of a normal gut microbiome in calves may help identify novel methods for enhancing gut health and production efficiency in cattle.

There is a growing market for organic food, including dairy and beef products. In the United States, products sold as organic must be produced under specific conditions, such as the exclusion of exposures to antimicrobial drugs (AMDs), anthelmintics, exogenous hormones, and chemicals such as fertilizers and pesticides. (8). Studying the development of the gut microbiome in calves raised on organic dairies provides a unique research opportunity to evaluate microbiome development in the absence of early-life exposure to AMDs and other exogenous inputs.

Early metagenomic studies revealed the gut microbiome’s complexity, but shallow sequencing limited insights into rare features, which can be as crucial as abundant ones. This applies to taxonomic features like bacterial species and genes, including antimicrobial resistance (AMR) genes, which can make up as little as 0.1%–0.01% of shotgun reads from feces (9, 10). Thus, our research group has demonstrated the value of deeper sequencing for the characterization of the resistome using target-enriched (TE) shotgun sequencing (9, 11–13).

The purpose of this study was to provide an in-depth characterization of the microbiome and resistome of feces during early life. Using 16S rRNA gene sequencing and TE shotgun sequencing for ARGs, we analyzed microbial communities in the feces of young Holstein calves of various ages, raised without antibiotics, hormones, or chemicals.

## MATERIALS AND METHODS

### Study overview

This cross-sectional study was conducted using 49 female Holstein heifers that were born and raised at a large USDA-certified organic dairy in northwestern Texas. Fecal samples were collected per rectum on a single day from 7 to 12 calves in each of the five different age groups. The communities of all bacteria (the microbiome) and all resistance genes (the resistome) identified in the fecal samples were characterized using 16S rRNA gene sequencing and target-enriched shotgun sequencing, respectively (12, 13). The fecal microbiome and resistome were deeply interrogated to characterize age-associated changes in calves of different ages, from birth to 99 days, in the absence of exposures to antimicrobial drugs, anthelmintics, hormones, or pesticides.

### Study population, animal management, and sample collection

Animals in this study were born and raised on a large USDA-certified organic dairy located in West Texas. Within 1 h of birth, calves received 4 L of colostrum and were removed from their dams when they were 12–24 h old. Female calves were raised until weaning in individual hutches with small enclosures spaced approximately 1 ft apart to minimize direct contact among animals and reduce the potential for the spread of disease. Young calves were fed 2 L of pasteurized organic milk twice daily and provided *ad libitum* access to an organic grain supplement and water. Calves were weaned when they were approximately 90 days old and housed in groups of six calves in a larger enclosure containing a larger group hutch; alfalfa hay, grain supplement, and water were provided *ad libitum*. Hutches were bedded with clean straw, which was periodically cleaned, and bedding was replaced.

On sampling day, producers provided a list of all female calves, and a random number table was employed to conduct stratified random sampling within the different age cohorts. Samples were collected from different calves in five age groups (no calves were sampled twice): early pre-weaning at 2–3 days old (Pre 1), late pre-weaning at 5 weeks old (Pre 2), just before weaning at 12–13 weeks old (Pre 3; individually housed), immediately after weaning at 12–13 weeks old (Post 1; group housing), and later post-weaning at 13–14 weeks old (Post 2; group housing; Table S1). The sampling plan was developed *a priori* to evaluate samples from calves of various ages, nutritional stages (milk-fed vs weaned), and housing arrangements (individual vs group). Calves enrolled in the study were restrained in their pens, and approximately 2 g of feces was collected per rectum using a clean exam glove that was changed between animals. Feces were placed in a sterile tube containing 2 mL of 100% ethanol to stabilize microbial communities (14), immediately cooled on ice, and transported to the laboratory within 2 h of sample collection, where they were stored at −80°C until further processing.

### DNA extraction

Genomic DNA was isolated from 100 mg of feces using a QIAamp PowerFecal DNA Kit (Qiagen, Hilden, Germany) according to the manufacturer’s protocol. Following isolation, DNA quantity was assessed using fluorometric quantification (Qubit dsDNA HS Assay; Thermo Fisher Scientific) to estimate double-stranded DNA concentration. For samples from the youngest calves (Pre 1), additional extraction and purification steps were performed when necessary to obtain sufficient DNA mass for library preparation, and products were combined. Feces collected from some of the youngest calves (i.e., 2 days old) were largely meconium, which required repeated extraction to obtain the target quantity of DNA. Despite repeated attempts, only trace amounts of DNA were isolated from three of these meconium-rich samples, and, as a result, they were excluded from downstream analyses regarding 16S rRNA gene sequencing and target-enriched shotgun sequencing. Two of those three samples had enough DNA to complete quantitative polymerase chain reaction analysis.

### 16S rRNA gene sequencing

The V3–V4 region of the 16S rRNA gene was amplified to prepare sequencing libraries using the 341f (5′-CCTACGGGNGGCWGCAG -3′) and 785r (5′-GACTACHVGGGTATCTAATCC-3′) primer pair (15). Amplification conditions included incubation at 98°C for 3 min, followed by 12 cycles of 98°C for 30 s, 55°C for 30 s, and 72°C for 1 min. Final elongation occurred at 72°C for 5 min. Amplicons were then purified using AMPure XP beads (Beckman-Coulter, Pasadena, CA, USA), and sequencing libraries were prepared with the Nextera IDT kit (Illumina, San Diego, CA, USA). Libraries were purified again using AMPure XP beads and pooled in equal proportions based on molarities. Library quality and fragment size distribution were evaluated using electropherogram analysis (Agilent TapeStation) to ensure that libraries met the manufacturer-recommended concentration and fragment size criteria for sequencing. Sequence was performed on an Illumina NovaSeq 6000 instrument using paired-end chemistry (2 × 250 bp) at the University of Colorado Anschutz Medical Campus’ Genomics and Microarray Core (UC-GMC).

### Target-enriched shotgun sequencing for ARGs

Target-enriched sequencing libraries were prepared using the SureSelect XT HS2 Reagent Kit for Illumina Paired-End Multiplexed sequencing (Agilent Technologies), with modifications (supplemental text in GitHub: https://github.com/Microbial-Ecology-Group/Manuscript-Mh_TE_validation/blob/main/Methods/VERO%20Target%20Enriched%20Single%20%26%20Double%20Capture.docx). A custom bait design targeting ARG sequences originally curated in the MEGARes version 1 database (16) was employed to enrich the sequencing libraries for ARG sequences. Library quality and fragment size distribution were evaluated using electropherogram analysis (Agilent TapeStation) to ensure that libraries met the manufacturer-recommended concentration and fragment size criteria for sequencing. Resulting libraries were pooled in equal molar amounts and sequenced on an Illumina NovaSeq 6000 instrument using paired-end chemistry (2 × 150 bp) at the UC-GMC.

### Quantitative polymerase chain reaction analysis

To evaluate total microbial abundance in samples, qPCR was used with primers specific for conserved regions adjacent to V3 and V4 of the 16S rRNA gene (15). Briefly, 20 μL aliquots containing 10 ng of extracted DNA and PCR reagents were prepared with final concentrations of 450 nM for each sample (*n* = 50). Each of these sample aliquots included 10 μL of Quantabio PerfeCTa SYBR Green FastMix, Low ROX 2× MasterMix (Quantabio), and 10 μM (0.9 μL/reaction) of IDT primers. Negative controls, containing only MasterMix and nuclease-free water, were evaluated on each plate. Standard curves were generated by serial dilutions (2 × 10^1^–2 × 10^6^ copies) of the 16S rRNA gene from *Mannheimia haemolytica* genomic DNA (17). Calculations of genome equivalents per microliter were conducted according to Owen et al. (18) and were run concurrently with samples to quantify bacterial load. Analyses were performed using the QuantStudio 3 real-time PCR system (Applied Biosystems, Thermo Fisher Scientific). UDG activation was carried out for 2 min at 50°C, followed by denaturation at 95°C for 10 min and 40 cycles of denaturation at 95°C for 15 s and annealing/extension at 50°C for 15 s, concluding with the melt curve stage at 95°C for 15 s, 58°C for 30 s, and 95°C for 1 s. Extractions from one sample in the Pre1 cohort lacked enough DNA to be included, and two samples failed amplification (*n* = 47).

### Bioinformatics

Demultiplexed 16S rRNA gene sequence reads were imported into QIIME2 version 2024.10 (19). Amplicon sequence variants (ASVs) were generated using DADA2 (20), which filtered reads for quality, removed chimeric sequences, and merged overlapping paired-end reads. Forward reads were trimmed at 17 bp and truncated at 249 bp. Reverse reads were trimmed to 21 bp and truncated at 236 bp. Taxonomy was assigned using a Naïve Bayes classifier trained on the SILVA version 138.2 SSU NR 99 database (21), where sequences were trimmed to include only the base pairs from the V3–V4 region bounded by the 341f/785r primer pair. Reads mapping to chloroplast and mitochondrial sequences were filtered from the representative sequences and ASV table using the “filter-seqs” and “filter-table” functions, and a midpoint-rooted phylogenetic tree was generated using the “q2-phylogeny” pipeline with default settings, which was used to calculate phylogeny-based diversity metrics.

Demultiplexed TE metagenomic sequence reads were processed using the AMR++ version 2 pipeline and the MEGARes version 2 resistance database (http://megares.meglab.org; (11, 16). Briefly, reads were trimmed and filtered for quality using Trimmomatic (22), and bovine host DNA was removed by aligning trimmed reads to the *Bos taurus* genome with BWA (23). Remaining reads were aligned to the MEGARes version 2 database with BWA, and gene sequences aligning to more than 80% of the reference nucleotide sequences of ARG accessions were included in downstream analysis. Reads aligning to ARGs that require specific single nucleotide polymorphisms to confer resistance were removed from downstream analyses.

### Statistical analysis

Unless specified otherwise, R version 4.4.3 (24) was used for statistical analysis of data. 16S rRNA (ASV count table) and AMR-TE (ARG count table) data were then imported separately into phyloseq (25) using the “import_biom” function. Metadata was imported using “import_qiime_sample_data” and merged with each count table into a phyloseq object. Richness and Shannon diversity values were calculated for all samples with phyloseq. ASV and ARG counts for each sample were then normalized using cumulative sum scaling (26), and beta-diversity was analyzed using generalized UniFrac distances for ASVs (27, 28) and Bray-Curtis dissimilarity distances for ARGs. For microbiome analyses, we calculated unweighted, weighted, and generalized UniFrac distances to evaluate phylogeny-informed community structure while assessing the relative influence of rare vs abundant taxa. For resistome analyses, Bray-Curtis (abundance-weighted) and Jaccard (presence/absence) dissimilarities were used because ARG features lack a shared phylogenetic structure and are more appropriately analyzed using ecological dissimilarity metrics. From these distances, non-metric multidimensional scaling (NMDS) was performed and plotted. A permutational multivariate analysis of variance (PERMANOVA) was used to test for statistically significant differences in community or ARG composition using the vegan (29) and pairwiseAdonis (30) packages. To ensure statistically significant differences were not the result of unequal dispersion of variability between groups, permutational analyses of dispersion (PERMDISP) were conducted for all significant PERMANOVA outcomes using the vegan package. Additionally, the RAs of ASVs and ARGs within each sample were calculated and plotted using phyloseq.

To evaluate whether fecal communities became more similar to the oldest calves’ communities, we performed a distance-based trajectory (“convergence”) analysis separately for the microbiome and resistome data sets. Pairwise dissimilarities were calculated on CSS-normalized feature abundances using Bray–Curtis (abundance-weighted) and Jaccard (presence/absence) distances for the resistome data set. For the microbiome data set only, we additionally computed phylogeny-informed distances using unweighted and weighted UniFrac.

Changes in community distances to Post 2 samples were assessed using nonparametric tests across Pre 1, Pre 2, Pre 3, and Post 1 groups (Kruskal-Wallis, followed by Benjamini-Hochberg-adjusted pairwise Wilcoxon tests). To test for overall temporal structuring of communities, we performed Mantel tests (Spearman; 999 permutations) comparing the dissimilarity matrix to a distance matrix derived from ordered time points (i.e., calf age categories).

Pairwise Wilcoxon rank-sum tests were performed with a Benjamini-Hochberg correction for multiple comparisons. Differences in beta-diversity were tested using pairwise PERMANOVA with a Benjamini-Hochberg correction for multiple comparisons and 9,999 permutations. Additionally, pairwise PERMDISPs were carried out for all statistically significant PERMANOVA outcomes using 9,999 permutations to test for differences in the variability of dispersions. Analysis of Compositions of Microbiomes with Bias Correction (ANCOM-BC) was completed at the Family (microbiome) and Mechanism level (resistome) to analyze differential abundance of taxa with bias-corrected observed abundances using the R package ancombc2 (31). ANCOM-BC was also utilized to complete multiple pairwise comparisons against a pre-specified group (Post 2) in a Dunnett’s type of test (32). Only results that passed sensitivity testing were included.

Estimates of total microbial abundance were obtained using the estimated number of copies of the 16S rRNA genes per milligram of feces. As meconium samples contained very little fecal material, 16S gene copy numbers were normalized to gene copies per milligram of feces. This estimation is based on the results from qPCR reactions, the mass of the sample used in extractions, and the quantity of DNA obtained from those extractions. Gene copies per milligram of feces were calculated by scaling qPCR copy numbers obtained from 10 ng of template DNA to the total DNA yield from extraction and normalizing by the mass of fecal material extracted.


$$Copies\;per\;mg\;of\;feces=\frac{\left(\frac{ng\;DNA\;from\;extraction}{10ng}\right)x\;copies\;from\;qPCR}{feces\;extracted\;\left(mg\right)}.$$

Analysis showed that abundance data were non-normal according to the Shapiro-Wilk test, so differences were assessed using the Kruskal-Wallis Test. All graphical plotting was conducted in R after importing data into the ggplot2 package (version 3.5.1), apart from UpSet plots, which were produced using the UpSetR package (version 1.4.0).

## RESULTS

### Sequencing results

**Fig 1:**
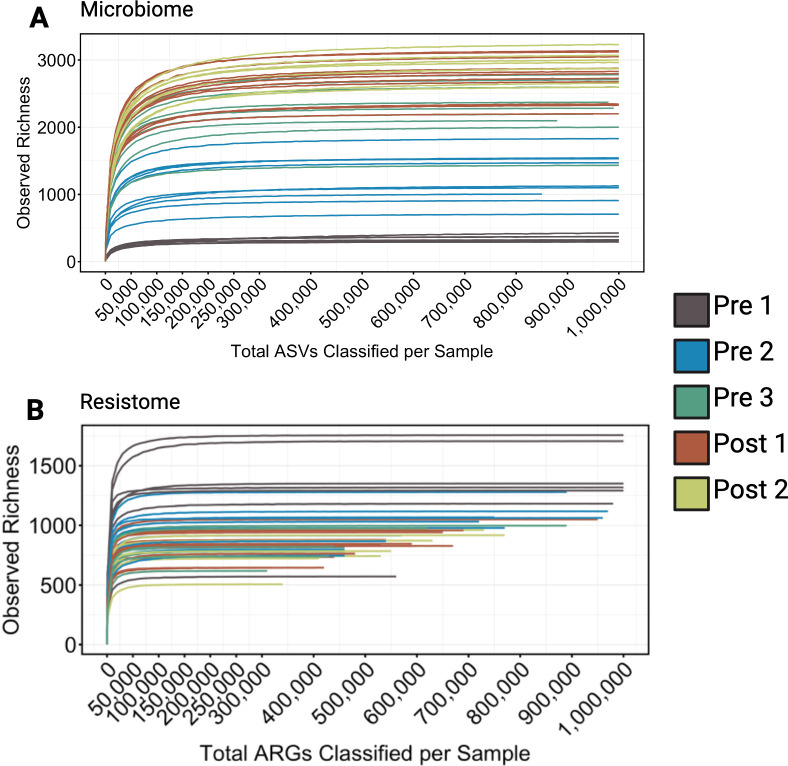
Rarefaction curves illustrating sequencing depth and feature richness across calf age cohorts for the microbiome and resistome. Panel **A** presents rarefaction curves for the microbiome based on 16S rRNA gene sequencing (classified ASVs), and panel **B** shows curves for the resistome based on target-enriched shotgun metagenomic sequencing (classified ARGs). Curves are grouped by five calf age cohorts: Pre 1 (2–3 days), Pre 2 (5 weeks), Pre 3 (12–13 weeks, pre-weaning), Post 1 (12–13 weeks, post-weaning), and Post 2 (13–14 weeks). For the microbiome, continued discovery of new features at sequencing depths beyond 100,000–200,000 reads highlights the necessity of deep sequencing to capture community diversity. In contrast, fewer than ~100,000 ARGs were detected per sample, reflecting lower feature richness in the resistome. Most curves reached a plateau, indicating that sequencing depth was sufficient to capture the majority of detectable features in both data sets.

The 16S amplicon sequencing yielded an average of 1,667,507 reads per sample (minimum reads per sample = 1,027,574, maximum = 2,727,936), with an average of 1,352,380 classified reads per sample (minimum = 853,965; maximum = 2,078,261). All samples had a sufficient number of ASVs for downstream analysis (Fig. 1A). Sequencing of TE shotgun libraries for the 46 samples produced an average of 60,502,240 reads each (minimum reads per sample = 27,125,512, maximum = 101,504,071). Bioinformatic analysis of the TE shotgun data yielded an average of 44,048,163 non-host reads per sample (minimum reads per sample = 20,888,997, maximum = 74,313,647). Of these, an average of 665,805 reads per sample were classified as ARG sequences (minimum = 314,043, maximum = 1,321,298). All samples also had sufficient numbers of reads classified as ARGs for downstream analysis (Fig. 1B). Rarefaction curves indicated that the sequencing depth was sufficient to comprehensively characterize the bacterial community using 16S rRNA and TE shotgun sequencing (Fig. 1). It is notable that ASV richness increased and did not plateau until reaching ≤250,000 classified ASVs. Nearly all ASVs (>99%) could be classified at the ranks of phylum, class, order, family, and genus ( Table S2). In comparison, ARG richness plateaued at approximately 100,000 classified ARGs (Fig. 1), which was achieved from approximately 30 million TE shotgun reads per sample (Fig. S1). Only about 0.1% of traditional shotgun reads from bovine feces might be classified as ARGs (10); however, this study’s TE sequencing showed an average of 1.1% of shotgun reads that could be classified as ARGs. Traditional shotgun sequencing would typically require substantially greater sequencing depth, approximately 300 million reads per sample, to achieve the same sequencing depth needed to fully explore the resistome.

### The microbiome and resistome follow opposing trends in richness, diversity, and shared ASVs/ARGs

**Fig 2:**
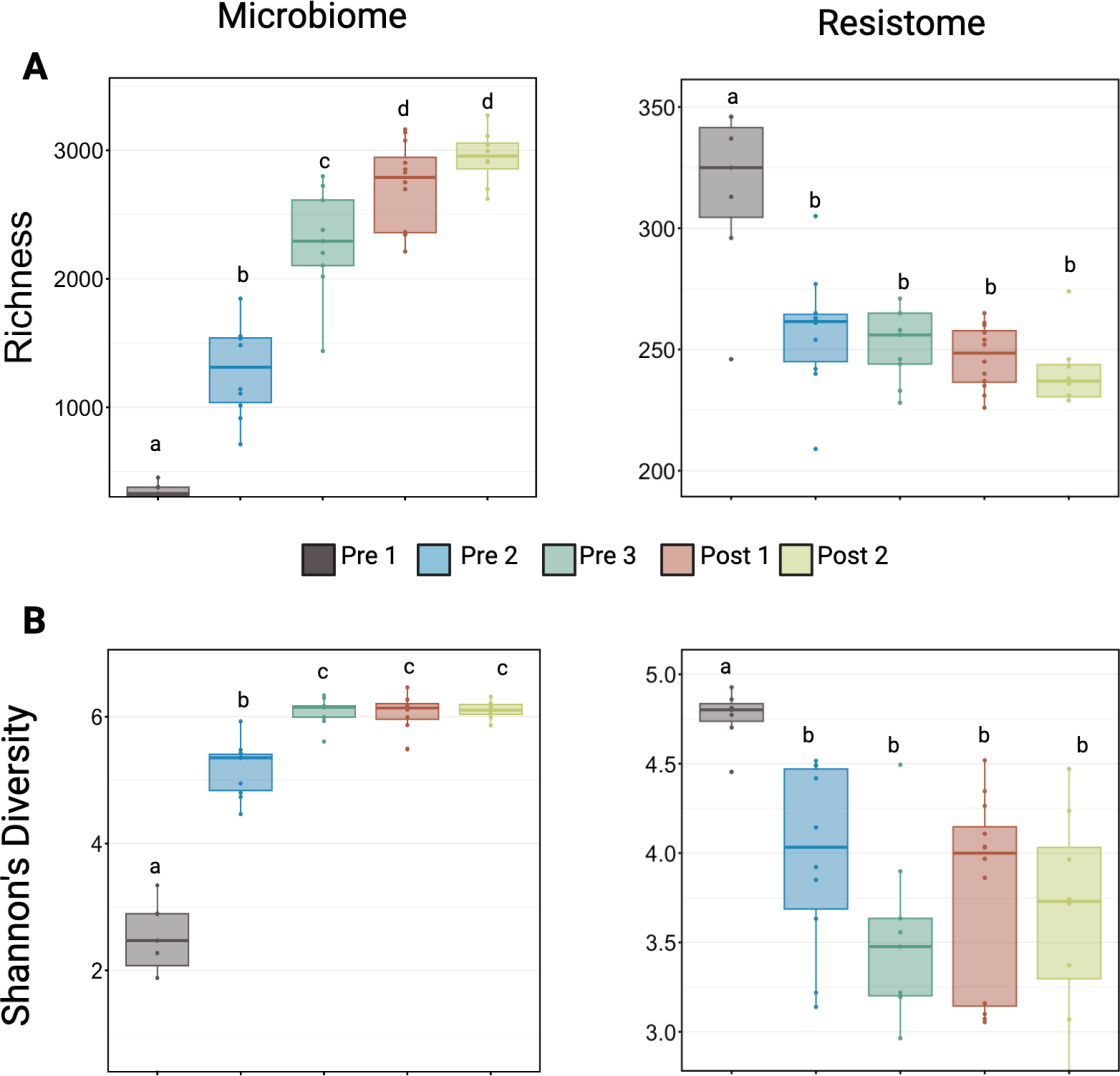
Richness and diversity of the fecal microbiome and resistome in dairy calves across five age groups. Boxplots show the (**A**) observed richness and the (**B**) diversity (Shannon’s diversity index) of amplicon sequence variants for the microbiome (left panels), and antimicrobial resistance genes of the resistome (right panels) across five developmental stages: Pre 1 (2–3 days), Pre 2 (5 weeks), Pre 3 (12–13 weeks, pre-weaning), Post 1 (12–13 weeks, post-weaning), and Post 2 (13–14 weeks). Richness and diversity of the microbiome increased significantly with age, stabilizing after weaning (Pre 3 to Post 2). In contrast, the resistome showed higher richness and diversity in the youngest calves (Pre 1) and decreased over time, stabilizing around 5 weeks of age (Pre 2). Different lowercase letters indicate statistically significant differences among groups (*P* < 0.05, Pairwise Wilcoxon rank-sum with Benjamini-Hochberg correction, *n* = 7–12).

**Fig 3:**
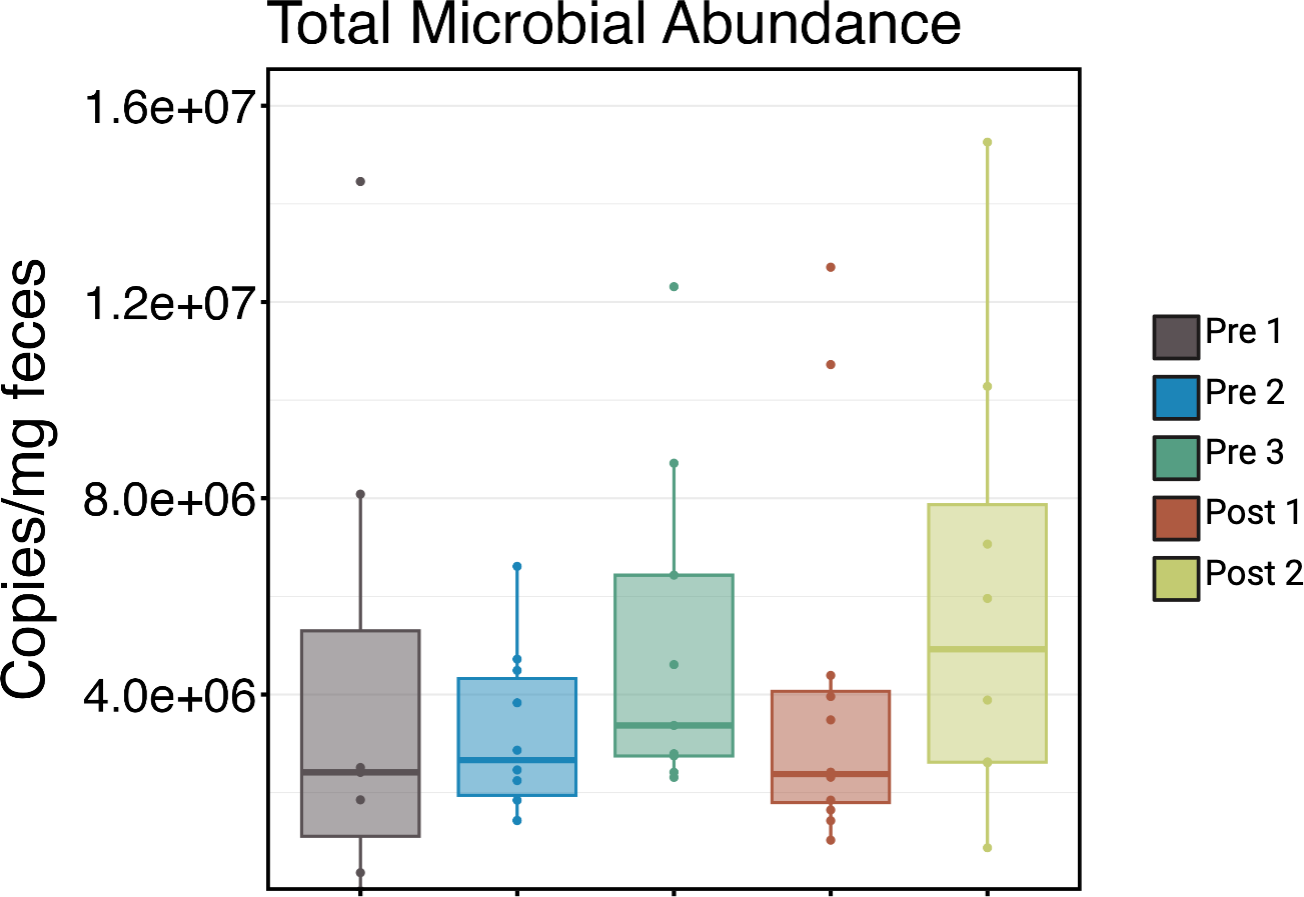
Total microbial abundance in calf feces across five age groups. Boxplot depicts total microbial abundance, measured as 16S rRNA gene copies per milligram of feces (via qPCR), in calves at five developmental stages: Pre 1 (2–3 days old), Pre 2 (5 weeks old), Pre 3 (12–13 weeks old, pre-weaning), Post 1 (12–13 weeks old, post-weaning), and Post 2 (13–14 weeks old). Variability was high in all age groups, and distributions were not statistically different among the cohorts (Kruskal-Wallis, *P* = 0.36, *n* = 7–12 per group).

The richness and diversity of microbial communities and ARGs changed significantly as calves aged, but in opposite directions. While microbial communities increased in both richness and diversity with age, the richness and diversity of ARGs decreased (Fig. 2). Interestingly, when analyzing estimated bacterial abundance through qPCR, the feces showed high variability at all age groups, ranging from undetectable to >1.2 × 10^7^ per mg of feces with distributions that broadly overlapped in all cohorts (Fig. 3). This, combined with the increasing richness, suggests that the changes in microbiome richness are not simply driven by bacterial load in the feces.

**Fig 4:**
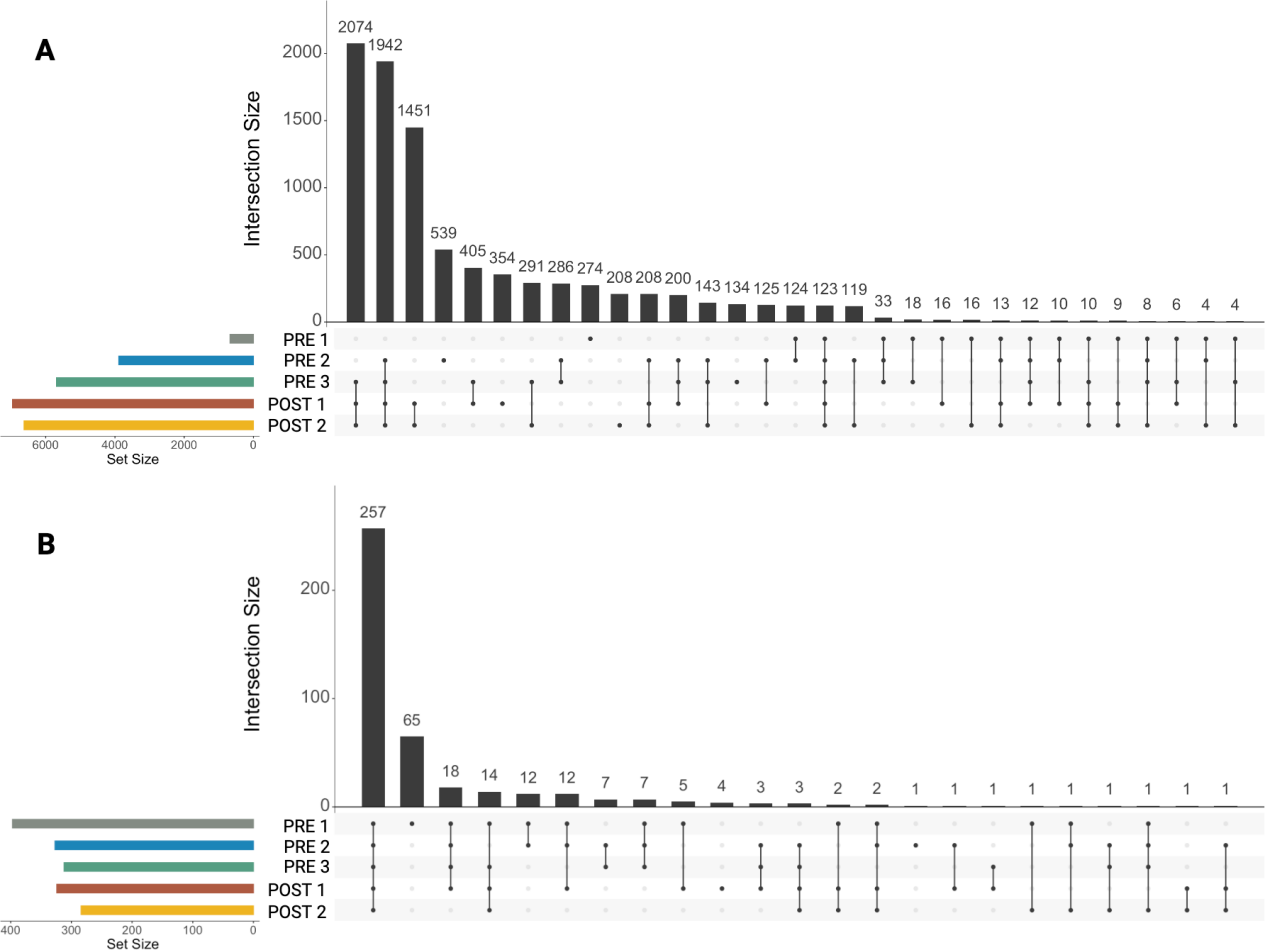
Unique and shared microbial taxa and ARGs among age groups. Upset plots illustrating the number of shared and unique features across five calf age cohorts for (**A**) microbial taxa based on 16S rRNA gene sequencing and (**B**) antimicrobial resistance genes identified through target-enriched shotgun sequencing: Pre 1 (2–3 days), Pre 2 (5 weeks), Pre 3 (12–13 weeks, pre-weaning), Post 1 (12–13 weeks, post-weaning), and Post 2 (13–14 weeks). Horizontal bars represent the total number of features detected in each age group, and vertical bars represent the size of intersections; the number of features shared across combinations of age groups, indicated by connected dots below each bar. ASVs or ARGs that were only in one or two samples were excluded from this analysis. The microbiome (**A**) exhibited greater total richness and more shared features across all five age groups compared to the resistome (**B**), which showed lower overall richness and fewer features shared among all groups.

The largest differences in richness existed between the two youngest age groups (Pre 1 vs Pre 2; 2–3 days vs 5 weeks), with microbial communities and ARGs exhibiting very large and statistically significant increases and decreases in richness and diversity, respectively (Fig. 2; Kruskal-Wallis, *P* < 0.05; pairwise Wilcoxon rank-sum test with Benjamini-Hochberg correction, *P* < 0.05; *n* = 7–12). ARG richness and diversity plateaued thereafter, with major features remaining similar around 5 weeks of age (Pre 2; Fig. 2; pairwise Wilcoxon rank-sum test with Benjamini-Hochberg correction, *n* = 7–12). This early apparent stabilization was further supported by analysis in UpsetPlot, which illustrated that calves of all ages share the majority of ARGs (Fig. 4B). After removing any ASVs or ARGs present in only one or two samples, calves of all ages in the study shared 257 ARGs (Fig. 4B). Interestingly, Pre 1 calves alone had the next largest number of unique ARGs (65) that were not shared with any other age group.

Diversity of microbial communities stabilized later than the resistome, around 12–13 weeks of age (Pre 3), just before weaning while still individually housed (Fig. 2B; Kruskal-Wallis, *P* < 0.05; pairwise Wilcoxon rank-sum test with Benjamini-Hochberg correction, *P* < 0.05; *n* = 7–12). Interestingly, richness appeared to stabilize slightly later, immediately after weaning (Post 1), when calves were in group housing. Calves of the same age, whether just before weaning in individual housing (Pre 3) or immediately after weaning in group housing (Post 1), displayed differing richness but similar diversity, suggesting that environmental factors may significantly influence community changes. Supporting the findings on diversity metrics with UpsetPlot, there were 2,037 ASVs shared among the older age groups (Pre 3, Post 1, and Post 2), with a decreasing number of shared ASVs as the calves aged, underscoring an opposing trend compared to shared ARGs (Fig. 4A).

### Early-life calves exhibit distinct microbial and resistome profiles, stabilizing at different rates with age

**Fig 5:**
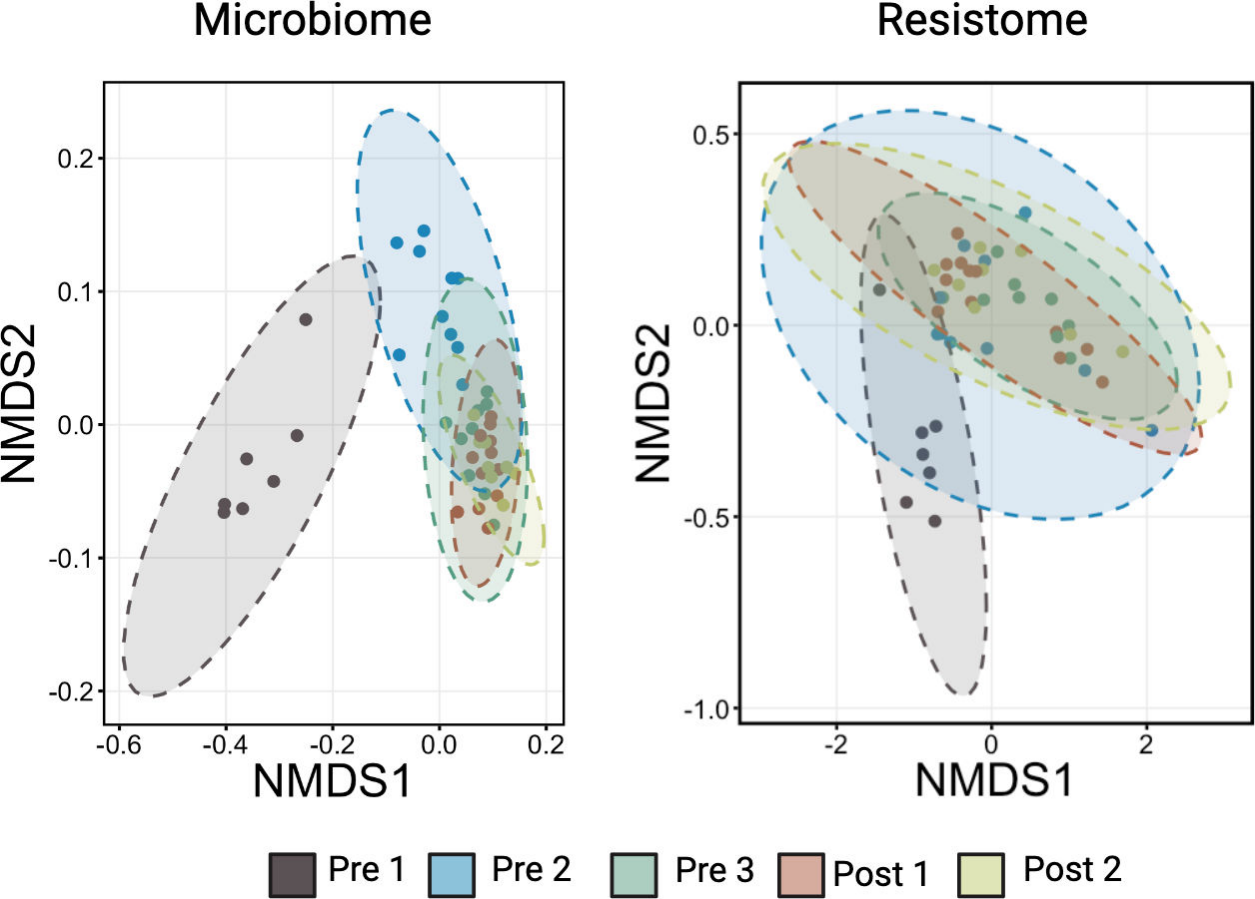
Ordination of fecal microbiome and resistome composition across calf age groups. NMDS plots based on generalized UniFrac distances for the microbiome community composition (left), while Bray-Curtis dissimilarity distances were used to characterize resistome composition (right) across five age groups: Pre 1 (2–3 days), Pre 2 (5 weeks), Pre 3 (12–13 weeks, pre-weaning), Post 1 (12–13 weeks, post-weaning), and Post 2 (13–14 weeks). Each point represents an individual calf sample, and colored ellipses represent 95% confidence intervals around group centroids. Microbiome composition shifted markedly with age, with distinct clustering observed between Pre 1 and older groups. Resistome composition also changed over time but stabilized earlier, with overlapping ellipses among older groups indicating less compositional divergence.

Statistical analysis of the generalized UniFrac and Bray-Curtis dissimilarity distances indicated that community structures of the microbiome and resistome differed significantly when comparing Pre 1 calves to all other age cohorts, with the resistome statistically stabilizing sooner than the microbiome, consistent with results observed in alpha-diversity metrics (Fig. 5; Table S2; PERMANOVA omnibus, *P* < 0.05; pairwise PERMANOVA with Benjamini-Hochberg correction, *P* < 0.05; *n* = 7–12). While the resistome composition remained similar across all other age groups from Pre 2 to Post 2, the microbiome continued to undergo structural changes until Post 1 and 2, when composition became stable (Fig. 5; Table S3 and S4; pairwise PERMANOVA with Benjamini-Hochberg correction, *P* < 0.05; *n* = 7–12). Tests for homogeneity of dispersion indicated no significant differences among age groups (PERMDISP, *P* > 0.50), confirming that observed PERMANOVA results reflected compositional differences rather than dispersion effects in both the microbiome and resistome.

**Fig 6:**
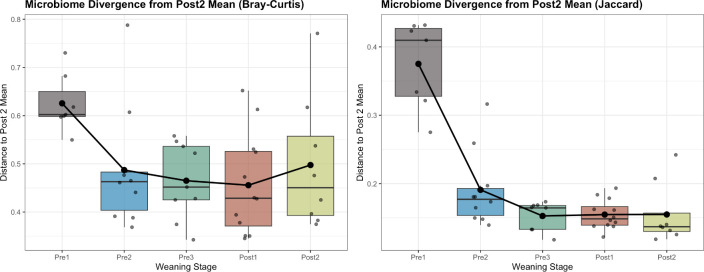
Distance-based trajectory (“convergence”) analysis of the fecal microbiome across age groups. Boxplots display the distribution of sample distances to the Post 2 centroid calculated from CSS-normalized feature abundances using phylogeny-informed metrics (weighted and unweighted UniFrac). Points represent individual samples, and the solid line connects group means to illustrate directional trends across ordered age groups. Microbiome composition differed significantly among age groups (Kruskal-Wallis, *P* < 0.0001). Pre 1 and Pre 2 samples remained significantly more dissimilar from Post 2 than later cohorts (pairwise Wilcoxon with Benjamini-Hochberg correction, *P* < 0.001), whereas Pre 3 and Post 1 samples did not differ significantly from Post 2 (*P* > 0.5), indicating progressive convergence toward a mature community structure by approximately 12–13 weeks of age. Mantel testing demonstrated a significant positive correlation between microbiome dissimilarity and age-group distance (generalized UniFrac; Spearman *r* = 0.50, *P* = 0.001), consistent with a directional and temporally structured pattern of microbial succession.

To evaluate whether fecal microbial communities exhibited directional convergence toward the mature community structure represented by the oldest calves (Post 2), we performed a distance-based trajectory analysis. For the microbiome, fecal samples from the youngest calves (Pre 1 and Pre 2) remained significantly dissimilar from Post 2 samples (Kruskal-Wallis, *P* < 0.0001; pairwise Wilcoxon, *P* < 0.001), whereas communities from Pre 3 and Post 1 calves were not significantly different from Post 2 (pairwise Wilcoxon, *P* > 0.5; Fig. 6), indicating progressive convergence toward a mature community structure with increasing age. Mantel testing further demonstrated a significant positive correlation between microbial community dissimilarity and ordered age-group distance (generalized UniFrac, Spearman *r* = 0.50, *P* = 0.001), indicating a directional and temporally structured pattern of microbial succession across early life time points. Comparable results were obtained using both weighted and unweighted UniFrac distances, indicating that convergence reflected shifts in both taxon presence–absence and relative abundance (RA).

**Fig 7:**
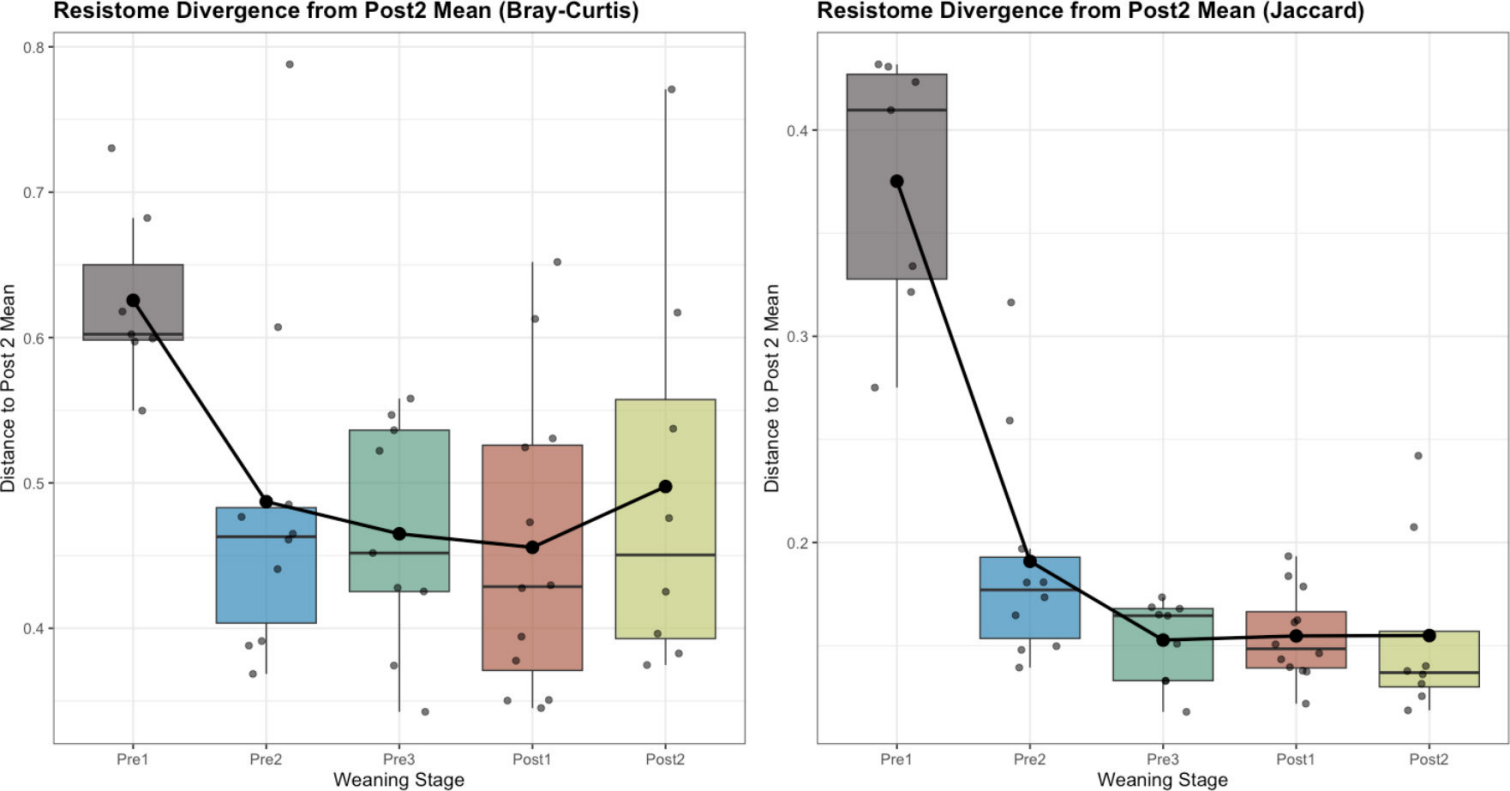
Distance-based trajectory (“convergence”) analysis of the fecal resistome across age groups. Boxplots display the distribution of sample distances to the Post 2 centroid calculated from CSS-normalized resistome feature abundances using Bray-Curtis (abundance-weighted) and Jaccard (presence/absence) dissimilarities. Points represent individual samples, and the solid line connects group means to illustrate directional trends across ordered age groups. Resistome composition differed among age groups for both Bray-Curtis (Kruskal-Wallis, *P* = 0.028) and Jaccard (*P* < 0.001) distances. Pairwise comparisons identified significant differences only between Pre 1 and Post 2 samples for Jaccard dissimilarity (pairwise Wilcoxon with Benjamini–Hochberg correction, *P* = 0.001), whereas significant pairwise differences were not detected using Bray-Curtis distances. Mantel testing demonstrated significant positive correlations between resistome dissimilarity and age-group distance for Bray-Curtis (Spearman r = 0.15, *P* = 0.003) and Jaccard (r = 0.41, *P* = 0.001), with the stronger association for Jaccard suggesting that early resistome changes were primarily driven by presence-absence turnover in resistance genes.

Succession analyses indicated that the resistome exhibited early turnover followed by relative stabilization. Overall differences among age groups were detected for both Bray-Curtis (*P* = 0.028) and Jaccard (*P* < 0.001) dissimilarities; however, pairwise comparisons revealed significant differences only between the youngest (Pre 1) and oldest (Post 2) calves when using Jaccard distances (pairwise Wilcoxon with Benjamini-Hochberg correction, *P* = 0.001; Fig. 7), with no significant pairwise differences observed using Bray-Curtis distances. These findings suggest that early-life variation in the resistome was driven primarily by presence–absence turnover rather than large shifts in dominant gene abundances. Mantel tests further revealed positive correlations between resistome dissimilarity and weaning-stage distance for Bray-Curtis (Spearman *r* = 0.15, *P* = 0.003) and Jaccard (*r* = 0.41, *P* = 0.001), with the stronger Jaccard relationship supporting a temporally structured pattern of early resistome succession driven primarily by gene turnover rather than changes in dominant gene abundance.

**Fig 8:**
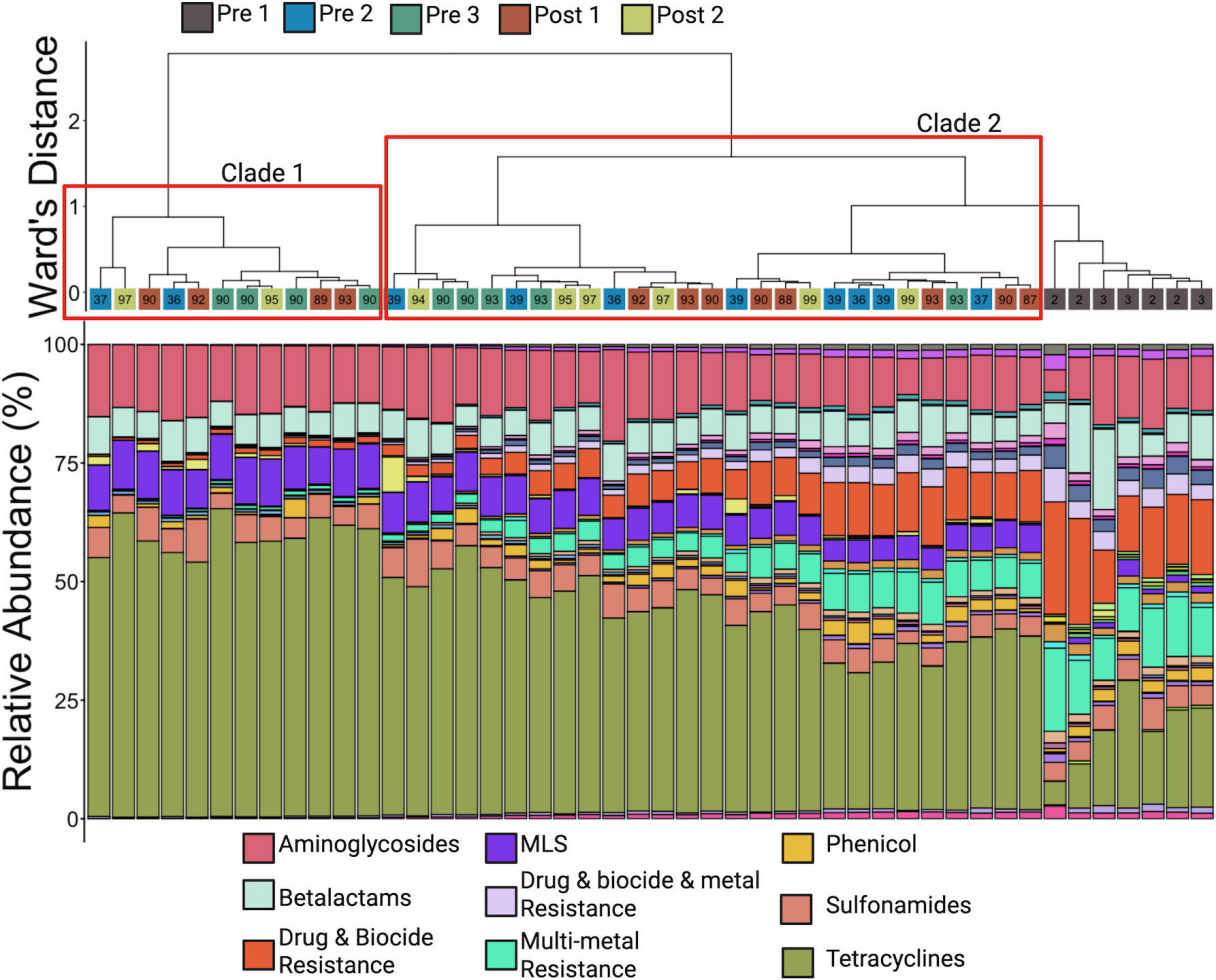
Hierarchical clustering and compositional profiles of fecal resistomes in dairy calves. The dendrogram evaluated the relatedness of resistome composition (top) generated using Ward’s method based on Bray-Curtis dissimilarity of ARG profiles, showing clustering of samples into two major clades (Clade 1 and Clade 2). Each sample is color-coded by age group: Pre 1 (2–3 days old), Pre 2 (5 weeks old), Pre 3 (12–13 weeks old, pre-weaning), Post 1 (12–13 weeks old, post-weaning), and Post 2 (13–14 weeks old). Bar plots (bottom) show the relative abundance of ARG classes within each sample. The nine most abundant classes are presented in the legend. Resistance genes conferring tetracycline resistance dominated across all samples but were more abundant in older calves. Younger calves (primarily in Clade 1) showed higher relative abundances of ARGs for aminoglycosides, beta-lactams, and drug–biocide resistance. Samples in Clade 2 had higher relative abundances of the metal-related resistance genes. These findings reflect both age-related changes and individual variation in resistome composition over time.

**Fig 9:**
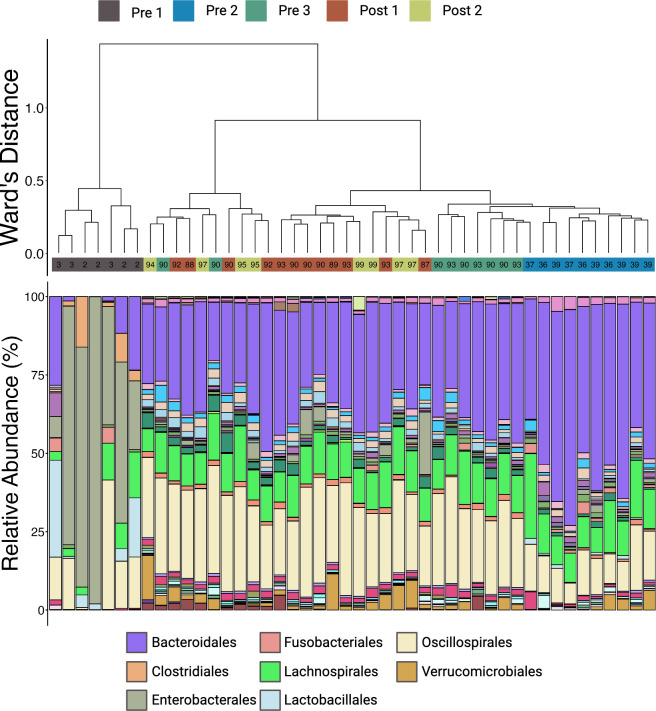
Hierarchical clustering and compositional profiles of dominant microbial orders in calf feces. The dendrogram generated using Ward’s method based on generalized UniFrac distances (top) evaluated the relatedness of microbial community composition across samples. Samples are color-coded by age group: Pre 1 (2–3 days old), Pre 2 (5 weeks old), Pre 3 (12–13 weeks old, pre-weaning), Post 1 (12–13 weeks old, post-weaning), and Post 2 (13–14 weeks old). Bar plots (bottom) show the relative abundance of bacterial orders identified from 16S rRNA gene sequencing. The eight most abundant orders are identified in the legend. Microbial communities of the youngest calves (Pre 1) were dominated by Enterobacterales, while older calves showed increased abundance of Bacteroidales, Oscillospirales, and Lachnospirales. These patterns illustrate a clear shift in community structure with age, consistent with the transition from a neonatal to a more mature gut microbiome.

Hierarchical clustering revealed that microbial and ARG communities from Pre 1 calves formed distinct clades. However, similar to the findings regarding alpha-diversity, hierarchical clustering of the Bray-Curtis dissimilarity data indicated that ARGs formed a sub-clade within a larger clade that includes most samples from other age groups (Fig. 8), while microbial communities remained more segregated. The microbial communities of Pre 2 calves formed their own respective clade, while a large clade containing the remaining communities consisted of three sub-clades (Fig. 9). One was primarily composed of Pre 3 calves, while the group-housed Post 1 and 2 calves exhibited intermixed communities (Fig. 9).

**Fig 10:**
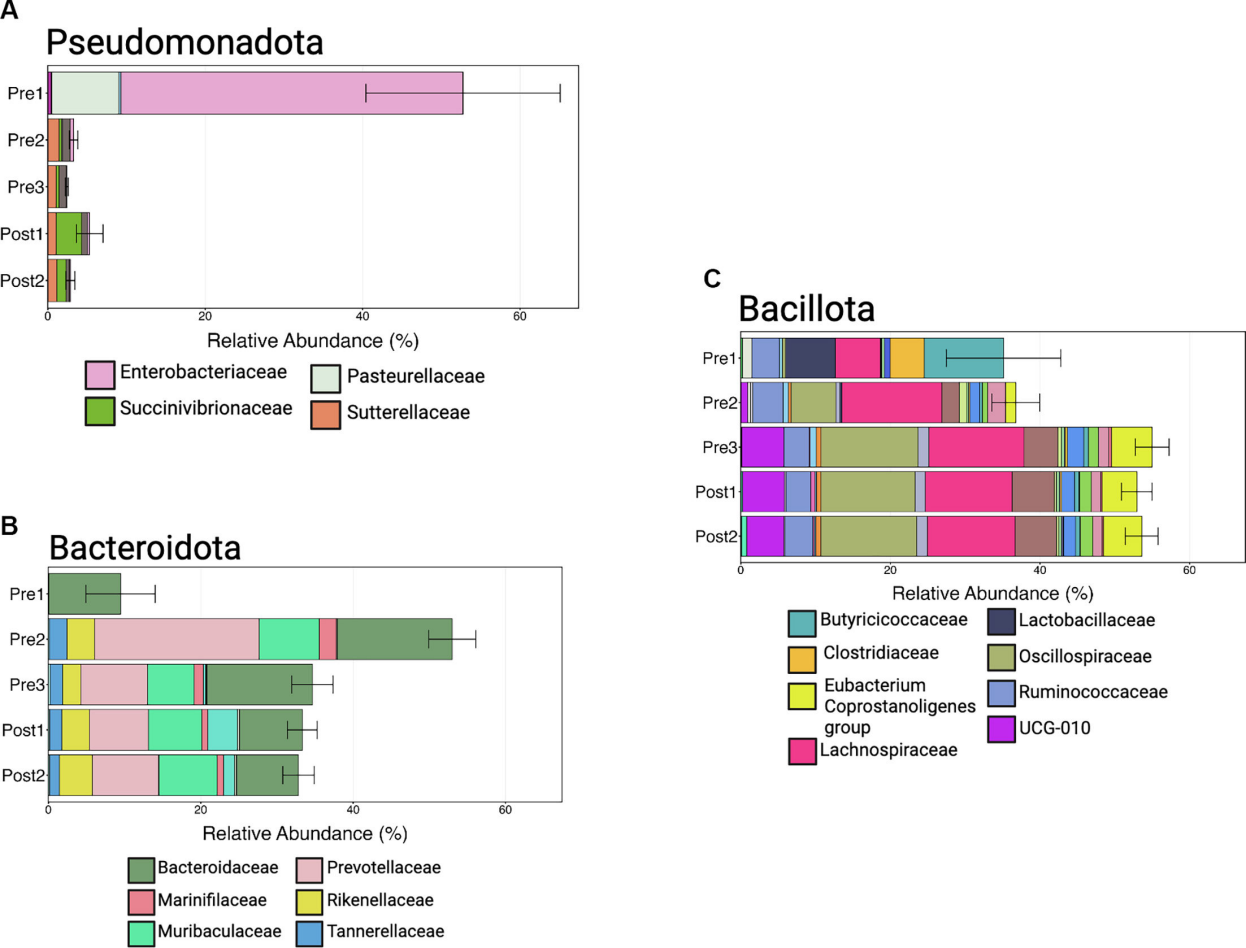
Taxonomic composition of dominant bacterial phyla and families across calf age groups. Mean relative abundances of major bacterial families within the phyla (**A**) Pseudomonadota, (**B**) Bacteroidota, and (**C**) Bacillota are shown for five developmental stages: Pre 1 (2–3 days), Pre 2 (5 weeks), Pre 3 (12–13 weeks, pre-weaning), Post 1 (12–13 weeks, post-weaning), and Post 2 (13–14 weeks). (**A**) Pseudomonadota were most abundant in the youngest calves (Pre 1), driven primarily by Enterobacteriaceae, and declined markedly with age. (**B**) Bacteroidota increased across age groups, with Bacteroidaceae and Muribaculaceae contributing substantially in later stages. (**C**) Bacillota increased with age, with greater relative abundance of families, including Lachnospiraceae, Oscillospiraceae, and Clostridiaceae in older calves. Bars represent mean relative abundance, and error bars indicate standard error of the mean. Patterns are consistent with age-associated shifts in gut community composition.

Visualization at the taxonomic rank of bacterial order illustrated that differences between the youngest calves (Pre 1) largely resulted from changes in the RA of Enterobacterales, which belongs to the phylum Pseudomonadota. Enterobacterales accounted for an average of 52.7% (±12.4% SE) in the Pre 1 calves but fell below 5% in older calves (Fig. 8; Table S5). Families within the phylum Pseudomonadota showed the largest decrease between calves aged 2–3 days (Pre 1) and older calves, driven by the family Enterobacteriaceae (Fig. 10A). In calves aged 2–3 days, members of Enterobacteriaceae represented 43.5% (±12.5% SE) of the entire microbial community but decreased to less than 1% in the other age groups (Fig. 10A; Table S5). Similarly, members of Pasteurellaceae displayed a marked decrease from calves aged 2–3 days (8.5% ± 8.2% SE) and remained below 1% in other cohorts (Table S5).

In the older age groups, Bacteroidales and Oscillospirales were the most abundant orders. The RA of these taxa increased with calf age, contrasting Pseudomonadota (Proteobacteria), and plateaued from the Pre 3 calves onward. Bacteroidales and Oscillospirales, belonging to Bacteroidota and Bacillota (Firmicutes), respectively, had families driving these changes. Within Bacteroidota, the RAs plateaued at 12–13 weeks of age while calves were still individually housed (Fig. 10B). This increase in Bacteroidota was largely due to shifts in the RAs of Muribaculaceae and Prevotellaceae. Muribaculaceae was less than 1% in Pre 1 and remained above 6% for all subsequent age groups (Fig. 10B; Table S5), while Prevotellaceae exhibited more variable changes, peaking in abundance for Pre 2 calves (21.5% ± 3.4% SE) and then decreasing to around 10% for Pre 3, Post 1, and Post 2 calves (Fig. 10B; Table S5). Bacteroidaceae maintained a relatively consistent abundance across all age groups, suggesting a potential role this taxon may play throughout the calves’ lives (Fig. 10B; Table S5).

Abundance of the phylum Bacillota remained stable as calves aged, but the families within the phylum underwent significant shifts, notably Butyricicoccaceae, Lachnospiraceae, and Oscillospiraceae. Butyricicoccaceae, particularly in calves aged 2–3 days, constituted 10.6% (±3.8% SE) of the RA and subsequently remained below 1% in all other age groups (Fig. 10C; Table S5). Members of Lachnospiraceae increased numerically from Pre 1 (6% ± 2.1% SE) to all other age groups, where the RA appeared stable (Fig. 10C; Table S5). Oscillospiraceae similarly increased between the two youngest age groups (Pre 1, 0.4% ± 0.2% SE; Pre 2, 6% ± 1% SE) and then made no more changes just before weaning (Fig. 10C; Table S5). Conversely, the RA of Lactobacillaceae decreased over time. The most substantial change was observed between calves aged 2–3 days (6.7% ± 4.5% SE) and those at 5 weeks of age (0.2% ± 0.1% SE; Table S5). Lactobacillaceae was virtually absent (<0.1%) in older calves (Fig. 10C; Table S5).

**Fig 11:**
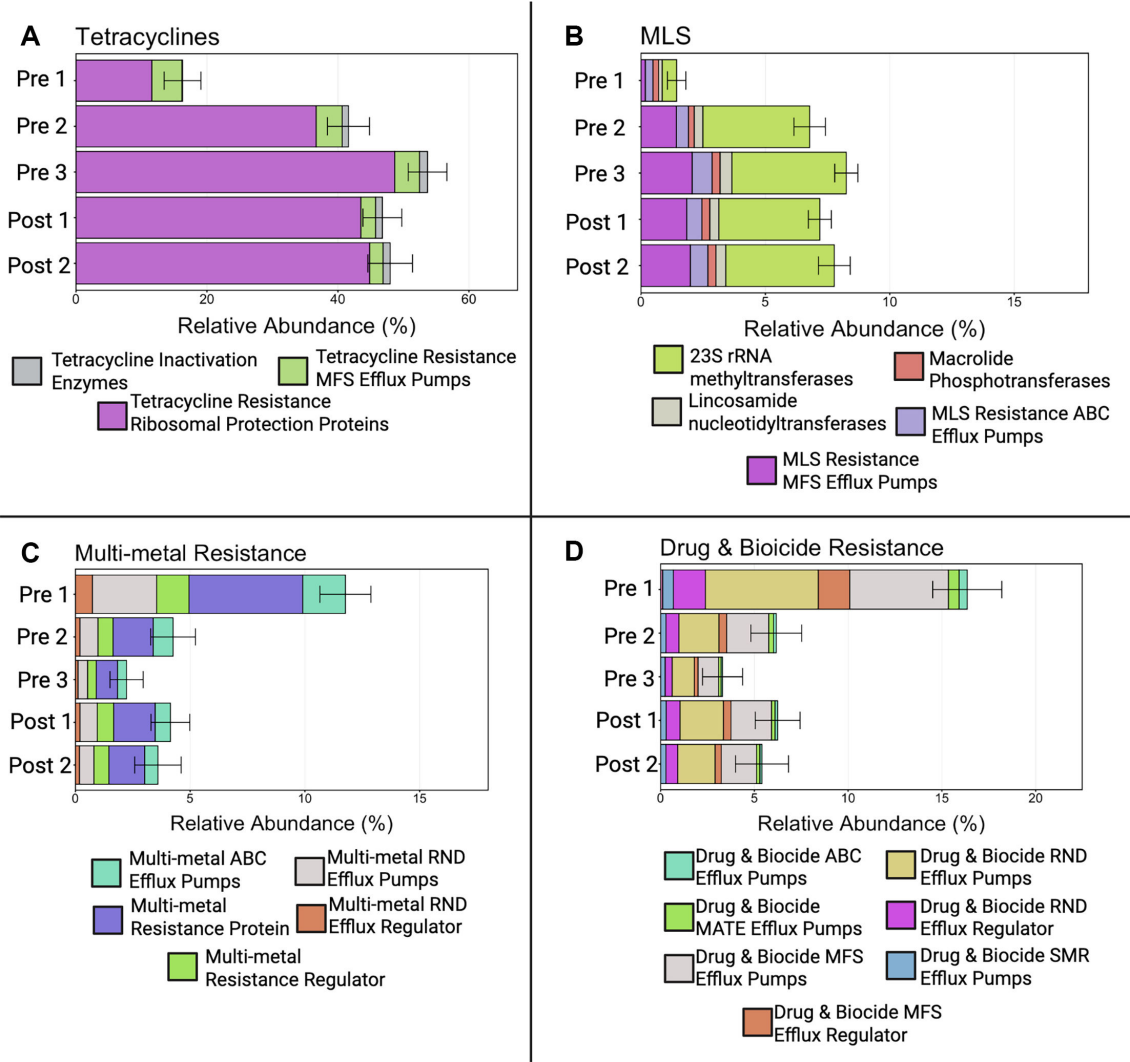
Relative abundance and mechanism-level composition of selected antimicrobial resistance gene classes across calf age groups. Mean relative abundances and associated resistance mechanisms are shown for ARGs conferring resistance to (**A**) tetracyclines, (**B**) macrolide–lincosamide–streptogramin drugs, (**C**) multi-metal resistance, and (**D**) drug and biocide resistance across five age groups: Pre 1 (2–3 days), Pre 2 (5 weeks), Pre 3 (12–13 weeks, pre-weaning), Post 1 (12–13 weeks, post-weaning), and Post 2 (13–14 weeks). (**A**) Tetracycline resistance genes increased with age and were primarily composed of ribosomal protection proteins and tetracycline efflux mechanisms. (**B**) MLS resistance genes, including 23S rRNA methyltransferases and associated efflux mechanisms, were minimal in the youngest calves and increased after 5 weeks of age. (**C**) Multi-metal resistance genes were most abundant in the youngest calves and declined with age, with contributions from multiple efflux and regulatory mechanisms. (**D**) Drug and biocide resistance genes also decreased over time and were composed of diverse efflux-associated mechanisms. Stacked bars represent the mean relative abundance of resistance mechanisms within each class, and error bars indicate the standard error of the mean. These patterns demonstrate age-associated shifts in both the abundance and composition of resistance gene classes during early-life community development.

Outside the clade containing the youngest calves, resistomes from all other age groups were largely intermixed but divided between two clades (Fig. 8). These two clades arise from differing RAs of ARGs conferring resistance to tetracyclines, which were higher in Clade 1 (Fig. 8, Clade 1), compared to those conferring resistance to metals more common in Clade 2 (Fig. 8). Visualization at the level of drug class reveals that the composition of the resistome is notably different in the youngest calves (2–3 days of age), where genes conferring resistance to tetracyclines are less abundant (Fig. 11A). This increase in older calves was primarily due to the RA of ribosomal protection proteins, which accounted for 11.6% (±2.7% SE) in Pre 1 calves and increased to 44.8% (±3.7% SE) in the oldest calves (Fig. 11A; Table S6). Likewise, genes conferring resistance to macrolide–lincosamide–streptogramin (MLS) were virtually undetectable in the resistome of Pre 1 calves but were among the most abundant ARGs found in older calves (Fig. 11B). Mechanisms within the MLS drug class driving this substantial change included increases in the RAs of 23S rRNA methyltransferases and MFS efflux pumps after the calves surpassed 2–3 days of age (Fig. 11B). In Pre 1 calves, 23S rRNA methyltransferases were less than 1% (±0.2% SE) and increased to 4.4% (±0.4% SE) by Post 2 (Fig. 11B; Table S6). The MFS efflux pumps associated with MLS resistance demonstrated a similar trend, also being less than 1% (±0.1% SE) for Pre 1, then reaching 2% (±0.1% SE) in the oldest calves (Fig. 11B). In contrast, genes conferring multi-metal resistance, along with those conferring drug and biocide resistance, exhibited a decrease in abundance after 2–3 days of age (Fig. 11C and D). The reduction in multi-metal resistance was mainly attributed to a decline in multi-metal resistance proteins and multi-metal RND efflux pumps after calves surpassed 2–3 days of age (Fig. 11C). Multi-metal resistance proteins decreased from 5% (±0.6% SE) to 1.5% (±0.4% SE) by Post 2, while RND efflux pumps initially at 2.8% (±0.1% SE) in Pre 1 calves remained below 1% for all other age groups (Table S6). The decline in drug and biocide resistance was largely due to MFS efflux pumps and RND efflux pumps decreasing in RA after calves were older than 2–3 days of age (Fig. 11D). MFS efflux pumps recorded at 5.2% (±0.7% SE) with RND efflux pumps at 6.0% (±1.2% SE) in Pre 1 calves, both dropping to around 2% in Post 2 calves (MFS efflux pumps at 1.9% ± 0.5% SE; RND efflux pumps at 2.0% ± 0.5% SE; Table S6).

### Differential changes in the calf gut microbiome and resistome

**Fig 12:**
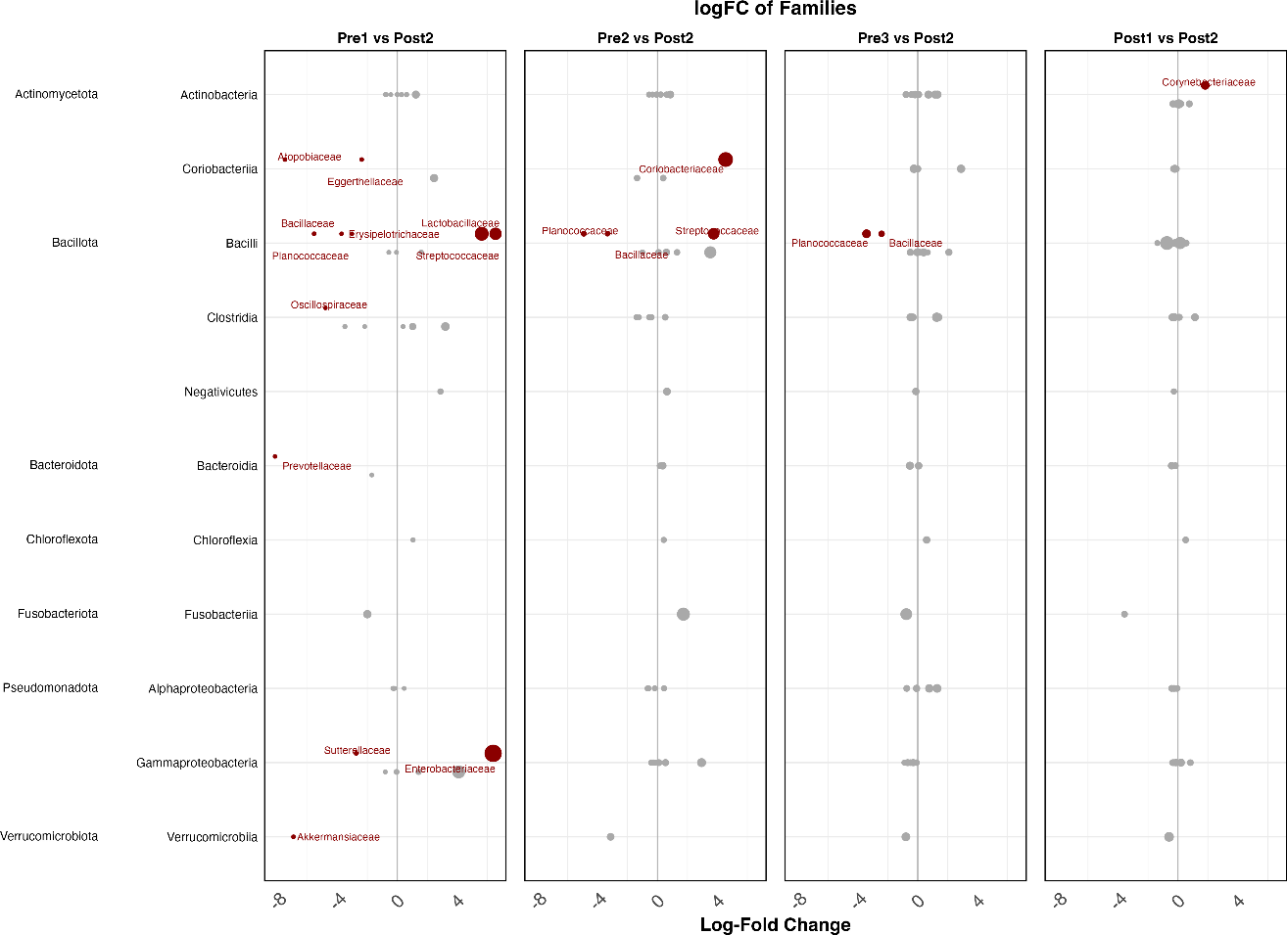
Differential abundance of bacterial families across calf age groups. Log_2_ fold-change plots showing differences in relative abundance of bacterial families in fecal samples across age groups: Pre 1 (2–3 days), Pre 2 (5 weeks), Pre 3 (12–13 weeks, pre-weaning), Post 1 (12–13 weeks, post-weaning), and Post 2 (13–14 weeks). The oldest age group (Post 2) served as the reference for all comparisons. Each point represents a bacterial family; red points indicate statistically significant differences (ANCOM-BC, *q* < 0.05), and point size reflects mean relative abundance. Positive Log_2_ fold-change values indicate enrichment relative to Post 2, whereas negative values indicate depletion. Early-life samples (Pre 1 and Pre 2) were enriched in families, including Enterobacteriaceae, Lactobacillaceae, and Streptococcaceae relative to Post 2, whereas several anaerobe-associated families, including Bacteroidaceae, Planococcaceae, and Oscillospiraceae, exhibited lower relative abundance in the youngest calves and increased with age. Fewer differentially abundant families were observed in later age groups, with minimal differences between Post 1 and Post 2. These patterns demonstrate progressive compositional shifts in the gut microbiota associated with maturation and the transition through weaning.

ANCOM-BC and a Dunnett’s test were used to evaluate potential changes in the abundance of families in the microbiome, using Post 2 as the reference, revealing differences unseen in RA plots. Log-fold changes of *Prevotellaceae, Anaerovoracaceae, Atopobiaceae, Erysipelotrichaceae, Oscillospiraceae, Eggerthellaceae, Sutterellaceae, Planococcaceae, Bacillaceae*, and *Akkermansiaceae* were all lower in the Pre 1 group compared to the Post 2 group (*q*-value < 0.01; Fig. 12; Table S7). Meanwhile, *Streptococcaceae, Lactobacillaceae,* and *Enterobacteriaceae* were higher (*q*-value < 0.01; Fig. 12; Table S7). As the calves aged, changes continued to occur. Pre 2 calves maintained higher levels of *Streptococcaceae* and experienced an increase in *Coriobacteriaceae* compared to the older calves (*q*-value < 0.01; Fig. 12; Table S7). *Anaerovoracaceae, Planococcaceae*, and *Bacillaceae* remained lower in this age group as well (*q*-value < 0.05; Fig. 12; Table S7). Pre 3 calves exhibited fewer changes, with lower Log_2_ fold changes observed only for *Planococcaceae* and *Bacillaceae* (*q*-value < 0.05; Fig. 12; Table S7). Interestingly, in the Post 1 group compared to the Post 2 group, an increase in *Corynebacteriaceae* was the only family exhibiting any changes and was absent in all other age groups (*q*-value = 0.01; Fig. 12; Table S7).

**Fig 13:**
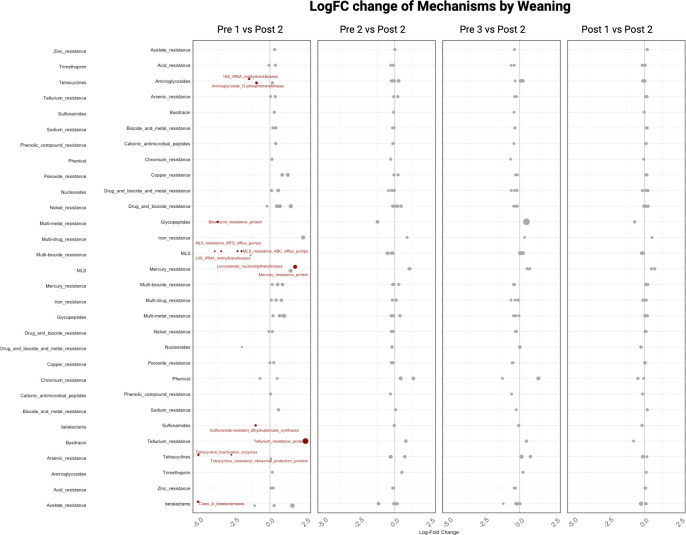
Differential abundance of antimicrobial resistance mechanisms across calf age groups. Log_2_ fold-change plots showing differences in the relative abundance of antimicrobial resistance mechanisms across age-group comparisons: Pre 1 (2–3 days), Pre 2 (5 weeks), Pre 3 (12–13 weeks, pre-weaning), and Post 1 (12–13 weeks, post-weaning), each relative to Post 2 (13–14 weeks), which served as the reference group. Each point represents a resistance mechanism; red points indicate statistically significant differences (ANCOM-BC, *q* < 0.05), and point size reflects mean relative abundance. Positive Log_2_ fold-change values indicate enrichment relative to Post 2, whereas negative values indicate depletion. The greatest number of differentially abundant mechanisms was observed in comparisons involving the youngest calves (Pre 1 vs Post 2), where multiple resistance mechanisms, including tetracycline ribosomal protection proteins, aminoglycoside-modifying enzymes, β-lactamases, and several efflux pump classes, were significantly lower relative to the reference group, while select metal resistance mechanisms were higher. In contrast, few significant differences were observed in comparisons among older age groups (Pre 2, Pre 3, and Post 1 versus Post 2), indicating that major shifts in resistome functional composition occurred early in life and remained relatively stable thereafter.

When analyzing the differential abundance of ARGs at the mechanism level, the majority of changes observed were between the Pre 1 cohort and the reference group (Post 2). There were significantly lower (*q*-value < 0.05) Log_2_FC in tetracycline resistance ribosomal protection proteins, tetracycline inactivation enzymes, sulfonamide-resistant dihydropteroate synthase, MLS MFS efflux pumps, MLS ABC efflux pumps, lincosamide nucleotidyltransferases, class D beta-lactamases, bleomycin resistance proteins, aminoglycoside O-phosphotransferases, and 23S and 16S rRNA methyltransferases. In contrast, tellurium resistance proteins and mercury resistance proteins were significantly higher (*q*-value < 0.05) in Log_2_FC compared to the reference group (Fig. 13; Table S8).

## DISCUSSION

Our results demonstrate the ubiquitous nature of AMR genes, which are found in abundance from early life in young calves, even in the absence of any AMD use on the farm. This highlights the natural occurrence of ARGs in all bacterial populations and aligns with observations in humans where ARGs can be present in individuals not directly exposed to antibiotics (33). Furthermore, this study shows that extremely large changes in the resistome composition are clearly driven by microbiome and host influences even without antimicrobial drug pressures. Previous research comparing calves from organic and conventional dairies has reported comparable numbers of unique ARGs regardless of antimicrobial exposure history (34), reinforcing the conclusion that ARGs are a normal feature in microbial communities and their presence, acquisition, and shifts in abundance are not solely dependent on antimicrobial drug exposures.

The sequencing depth achieved in this study enabled robust characterization of the fecal resistome and microbiome of young calves, allowing detection of both dominant and low-abundance community features. Previous studies in young calves have reported variable sequencing depths to characterize the microbiome and resistome (from 40,000 to 70,000 reads per sample for 16S sequencing and 40 million traditional shotgun reads to characterize ARGs [6, 35–37]), which were significantly lower than what was used in this study. Rarefaction of our 16S sequencing data indicated that depths of 200,000–250,000 for classified ASVs are more ideal to fully characterize the fecal microbiome. Similarly, it would require 100,000 reads classified as ARGs per sample to fully characterize the resistome, which would likely require an average of at least 100M non-host reads from traditional shotgun sequencing, or as many as 300M raw shotgun reads per sample (Fig. S1), depending on the amount of host DNA in samples (9, 10).

The fecal microbiome and resistome of neonatal calves (2–3 days old; Pre 1) are different from older calves, with both communities changing before statistically stabilizing by 12–13 weeks of age, with the microbiome remaining similar after weaning (group housing), and the resistome before weaning (individual housing). Taxonomic succession that was observed, in which the feces of very young calves are dominated by Pseudomonadota, whereas Bacillota and Bacteroidota are more prevalent in older calves, has also been observed by others (38). Stabilization of microbial community compositions, as assessed by generalized UniFrac and PERMANOVA, was not observed until after weaning when calves were housed as groups (Post 1), highlighting the potential influence of environmental and social exposures on the gut microbial development (Tables S13 and S4). Interestingly, other research has found that the shift to a dominance of Bacillota and Bacteroidota occurs prior to 5–7 weeks of age, and that the RA of these taxa is not influenced by weaning, at least at the phylum level at which previous data were reported (39, 40). In contrast, major features of the resistome appeared to stabilize earlier by 5 weeks of age (Pre 2), suggesting that ARGs may be primarily carried by microbial taxa that colonize the gut early in life. For example, genes conferring resistance to tetracyclines became predominant and reached abundances typical of adult cattle by the time calves were 5 weeks old.

Our findings support a model in which younger calves possess a richer and more diverse resistome than older animals (6, 35). However, the richness and diversity of the fecal microbiome followed an opposite trend, increasing as calves aged. This inverse relationship implies that early-dominant microbial taxa may harbor a greater repertoire of ARGs than those that predominate later in development. Culture-based studies report richer resistomes in young animals, though such methods can overrepresent resistance to specific drug classes like tetracyclines and ceftiofur (41). In our study, the RA of tetracycline resistance genes increased with age, contrasting with culture-based studies that reported the highest levels in the youngest calves (35). These discrepancies underscore the limitations of culturing approaches and highlight the advantages of TE shotgun sequencing in capturing the full complexity of the resistome.

One possible mechanistic explanation for this inverse relationship is ecological succession in the developing gut. Our analyses demonstrated clear directional compositional changes with age and evidence of taxonomic turnover, supporting a succession-based framework for early-life microbial and resistome development. Early in life, the calf gastrointestinal tract is colonized by facultative anaerobes and other pioneer taxa that often possess large and diverse accessory genomes, including mobile antimicrobial resistance determinants. These early colonizers may therefore establish a relatively diverse resistome despite low overall microbial diversity. As the gut environment matures and becomes more anaerobic, obligate anaerobes and metabolically specialized taxa associated with a stable rumen and hindgut environment increase in relative abundance. Competitive ecological processes and niche specialization among these later colonizers may reduce the diversity of resistance genes while increasing taxonomic richness and evenness of the microbiome. Maternal and environmental microbial inputs, including those derived from colostrum and early-life exposures, may also contribute to shaping the initial resistome and subsequent community succession. While the present study provides evidence consistent with this succession-based model, additional longitudinal and functional studies will be required to directly evaluate the mechanisms linking microbial community assembly and resistome maturation in young calves.

Age-related shifts in community composition were especially pronounced between the youngest calves (Pre 1) and all other groups. While previous studies suggest that microbial communities in cattle begin to resemble those of adults when they reach 8 and 11 weeks of age (1, 5), our data indicate that stabilization of the microbiome appeared to occur later, at around 12–13 weeks of age. Mantel testing confirmed that early-life communities differ and converge toward a stable structure. The timing of the stabilization may reflect differences in study design and housing conditions, as PERMANOVA confirmed significant differences in microbial structure until the transition to group housing at weaning. In contrast, apparent stabilization of the resistome when calves reached 3–7 weeks of age that was observed here is consistent with earlier findings (6). Succession analysis having the stronger Jaccard relationship suggested early-life variation is driven primarily by presence–absence changes, reflecting turnover in resistance gene presence/absence while dominant features stabilize. This may indicate that early colonizers have a diverse resistome that is not influenced by antibiotics, but the taxa carry a broad range of resistance organisms. As they age, the succeeding colonizers have fewer resistance genes.

Specific taxa and ARGs of clinical relevance showed dynamic changes in early life. Enterobacteriaceae were dominant in the youngest calves and declined with age, consistent with reports identifying these taxa as early colonizers and dysbiosis markers (1, 5, 36, 42). Conversely, the increase in members of the phylum Bacteroidota and tetracycline resistance genes with age may reflect the expansion of specific lineages such as Bacteroidaceae, which are known reservoirs of these genes (6). Similarly, the emergence of Muribaculaceae and MLS resistance genes at approximately 5 weeks of age coincided temporally, suggesting that members of this lineage may represent an important reservoir for MLS resistance determinants in the developing calf gut. Muribaculaceae has been shown to be among the most abundant taxa in ruminants, and its abundance was positively correlated with the abundance of erm-group ARGs and the overall abundance of MLS genes (13). Genomic analyses have demonstrated that Muribaculaceae frequently encode MLS resistance genes (43), supporting this interpretation while indicating the need for future work to directly link these determinants to specific taxa within the calf gut ecosystem.

The increase in tetracycline resistance genes with age despite the absence of antimicrobial exposure suggests that ecological rather than therapeutic factors may contribute to the dominance of this mechanism. One possibility is that tetracycline resistance determinants are carried by bacterial taxa that expand as the gut matures, resulting in increased relative abundance of these genes as community composition shifts. Alternatively, tetracycline resistance genes may be linked to mobile genetic elements or other fitness-associated traits that confer advantages unrelated to antimicrobial exposure, allowing them to persist and proliferate within the developing gut ecosystem. These findings highlight the importance of ecological and functional drivers of resistome composition beyond direct antimicrobial selection and warrant further investigation through longitudinal and genomic analyses.

Beyond describing compositional change, the observed trajectories have important implications for gut ecological development and antimicrobial resistance dynamics in young calves. Early-life communities were characterized by low microbial richness and dominance of facultative taxa, features typically associated with reduced ecological stability and increased susceptibility to perturbation. Such communities may be more vulnerable to dysbiosis or colonization by opportunistic organisms due to limited metabolic redundancy and niche occupancy. As calves aged, increasing microbial richness and expansion of obligate anaerobes were consistent with maturation toward a more functionally stable and resilient gut ecosystem. In parallel, the early-life enrichment and subsequent restructuring of the resistome suggest that neonatal calves may experience a period of greater resistome diversity and potential genetic fluidity, even in the absence of antimicrobial exposure. As community structure becomes more complex and specialized, declining resistome diversity may reflect reduced carriage or persistence of mobile resistance determinants. Together, these findings indicate that early-life microbial assembly represents a critical developmental window influencing both gut ecosystem resilience and the long-term architecture of resistance gene reservoirs in cattle production systems.

A notable limitation of this study is its cross-sectional design, as a longitudinal approach would allow better control of calf-level confounding factors and provide stronger inference regarding the temporal development of the microbiome and resistome. Additionally, this study was conducted within a single production system, which may limit the extrapolation of these findings to calves raised under different management conditions. However, performing the study within a single premises with consistent management across calves also provided important control for factors known to influence microbiome development (e.g., disease status, colostrum management, antimicrobial use, probiotics, and feeding strategies). This consistency allowed clearer interpretation of age-associated changes in the microbiome and resistome while minimizing variability introduced by differences in management or environment (44). In this study, the resistome appeared to stabilize prior to the switch from individual to group housing, suggesting that early colonizers strongly influence the establishing ARG profile. Consistent with this interpretation, ANCOM-BC analysis identified minimal changes in ARG mechanisms between pre- and post-weaning (i.e., between Pre 3 and Post 1 groups), suggesting major resistome shifts likely occur earlier in life. Finally, DNA re-extraction from feces (meconium) of the youngest calves was necessary to obtain the mass of DNA needed for the preparation of sequencing libraries. However, this process could potentially introduce minor bias when making comparisons to other samples; optimizing extraction methods for low-biomass neonatal fecal samples should be considered in future research.

In addition, future research should also include longitudinal sampling and experimental manipulation of environmental exposures to better understand factors driving ARG acquisition and persistence in young calves. This includes the investigation of the impact of common antimicrobial treatments. These findings provide a foundation for evaluating how early-life microbial and resistome changes with age may influence calf health and productivity and inform antimicrobial stewardship strategies in dairy production systems.

## Data Availability

All sequencing reads are available through BioProject PRJNA1438093 at the NCBI's Sequence Read Archive; accessions SAMN56529137 - SAMN56529183 (n = 47) were analyzed for this manuscript. The code and instructions for the bioinformatic and statistical analyses can be found at DOI https://doi.org/10.5281/zenodo.19139795. Customized methods used for target-enriched sequencing of Mh and antimicrobial resistance genes can be downloaded from DOI https://doi.org/10.5281/zenodo.19169706.

## SUPPLEMENTAL MATERIAL

Supplemental material spectrum.02510-25-s0001.pdf Figure S1 and Tables S1 to S8.

ASM does not own the copyrights to Supplemental Material that may be linked to, or accessed through, an article. The authors have granted ASM a non-exclusive, world-wide license to publish the Supplemental Material files. Please contact the corresponding author directly for reuse.
