# Supplementary material for: Early-life development of the microbiome and resistome in antibiotic-naïve dairy calves
Source: Microbiol Spectr. 2026 Apr 30;14(6):e02510-25. doi: 10.1128/spectrum.02510-25 (PMC13228081; doi:10.1128/spectrum.02510-25)
Supplement: Supplemental material — Figure S1 and Tables S1 to S8. [file spectrum.02510-25-s0001.pdf]

SUPPLEMENTAL MATERIAL

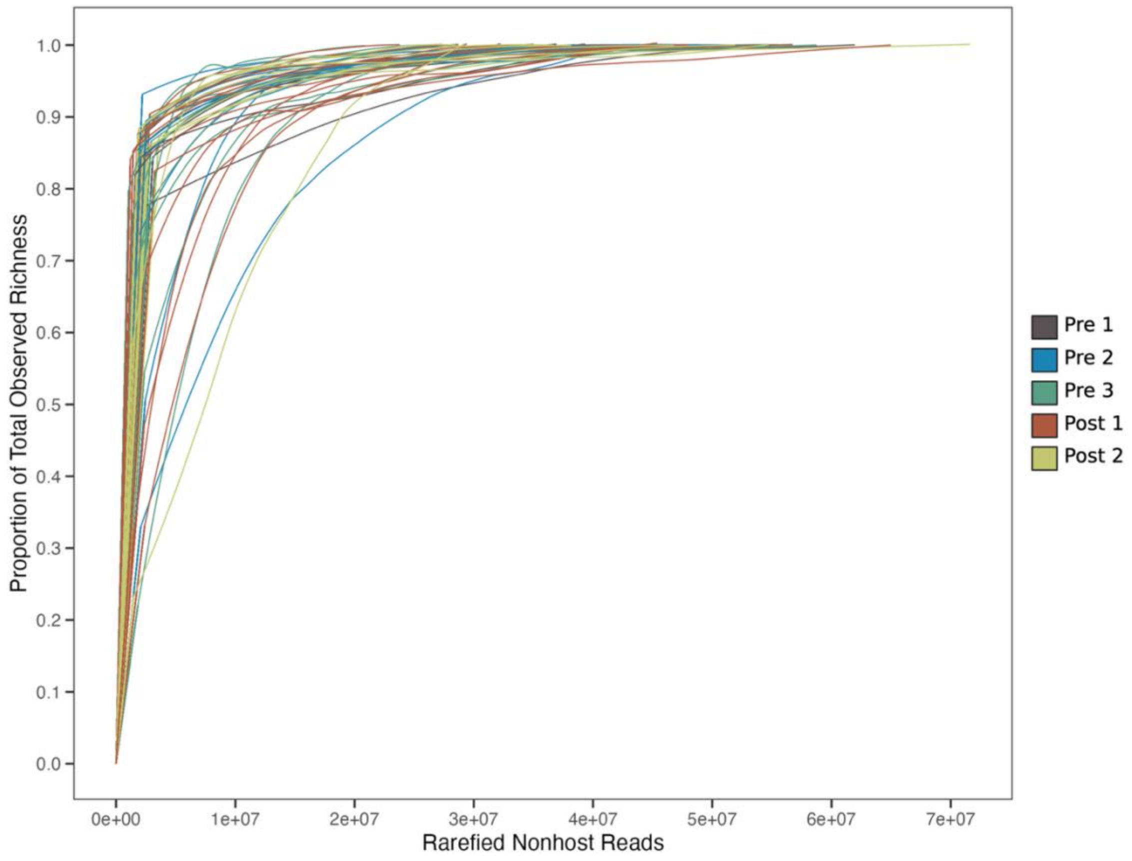

Supplementary Figure S1. Rarefaction analysis illustrating the proportion of total observed antimicrobial resistance gene (ARG) richness relative to sequencing depth (rarefied non-host reads) from target-enriched (TE) shotgun sequencing. Samples are colored by calf age cohort: Pre 1 (2-3 d-of-age), Pre 2 (5 wks-of-age), Pre 3 (12-13 wks-of-age, pre-weaning), Post 1 (12-13 wks-of-age, post-weaning), and Post 2 (13-14 wks-of-age). ARG richness plateaued at approximately 100,000 classified ARGs (Fig. 1), which was achieved from approximately 30 million TE shotgun reads per sample as illustrated here.

Supplementary Table S1. Description of calves and their groups.

Group Name	Group Description	Age (d)	Number of Calves in group
Early Pre-Weaning (Pre 1)	Housed in individual hutches	2-3	7
Later Pre-Weaning (Pre 2)	Housed in individual hutches; 5 wks-of-age;	36-39	10
Immediately before Weaning (Pre 3)	Calves were housed in individual hutches; 12-13 wks-of-age	90-93	9
Immediately after Weaning (Post 1)	Moved to group housing; 12-13 wks-of-age	87-93	12
Later Post-Weaning (Post 2)	Group housing; 13-14 wks-of-age	94-99	8

Abbreviations: wks = weeks; d = days.

Supplementary Table S2. Proportion of 16S rRNA gene sequence amplicon sequence variants (ASVs) that were classified at each taxonomic rank.

**Taxonomic Rank**					
	Phylum	Class	Order	Family	Genus
ASVs classified	100%	99.99%	99.68%	99.59%	99.91%

Supplementary Table S3. PERMANOVA and PERMDISP results from comparisons between microbial communities associated with the different age group cohorts (Pre 1 (2–3 days old), Pre 2 (5 weeks old), Pre 3 (12–13 weeks old, pre-weaning), Post 1 (12–13 weeks old, post-weaning), and Post 2 (13–14 weeks old). These were based on generalized UniFrac values. Significant results are bolded (p-adj. < 0.05).

**Test**	**Df**	**SS**	**Psuedo-F**	R^2	**p-adj.**	**PERMDISP (p-adj.)**
Overall	4	0.90	11.57	0.53	**0.001**	0.14
Pre 1 v. Pre 2	1	0.37	18.14	0.55	**0.0001**	0.07
Pre 1 v. Pre 3	1	0.45	25.57	0.65	**0.0004**	0.36
Pre 1 v. Post 1	1	0.52	29.16	0.63	**0.0002**	0.19
Pre 1 v. Post 2	1	0.46	25.13	0.66	**0.0002**	0.32
Pre 2 v. Pre 3	1	0.12	6.00	0.26	**0.0001**	0.09
Pre 2 v. Post 1	1	0.19	9.34	0.32	**0.0001**	0.06
Pre 2 v. Post 2	1	0.16	7.67	0.32	**0.0001**	0.17
Pre 3 v. Post 1	1	0.04	1.94	0.09	**0.0002**	0.67
Pre 3 v. Post 2	1	0.03	1.76	0.10	**0.0073**	0.78
Post 1 v. Post 2	1	0.02	0.87	0.05	0.74	0.97

Abbreviations: wks = weeks; d = days; v = versus; Df = degrees of freedom; SS = sum of squares; p-adj = adjusted p-value

Supplementary Table S4. PERMANOVA and PERMDISP results from comparisons between antimicrobial resistance genes associated with the different age group cohorts Pre 1 (2–3 days old), Pre 2 (5 weeks old), Pre 3 (12–13 weeks old, pre-weaning), Post 1 (12–13 weeks old, post-weaning), and Post 2 (13–14 weeks old). These were based on Bray-Curtis dissimilarity distances. Significant results are bolded (p-adj. < 0.05).

**Test**	**Df**	**SS**	**Psuedo-F**	R^2	**p-adj.**	**PERMDISP (p-adj.)**
Overall	4	1.89	5.5	0.35	**0.001**	0.95
Pre 1 vs Pre 2	1	0.81	8.48	0.36	**0.00075**	0.83
Pre 1 vs Pre 3	1	1.46	18.4	0.57	**0.0007**	0.64
Pre 1 vs Post 1	1	1.12	13.1	0.43	**0.0007**	0.87
Pre 1 vs Post 2	1	1.06	10.7	0.45	**0.0007**	0.88
Pre 2 vs Pre 3	1	0.23	2.90	0.15	0.15	0.40
Pre 2 vs Post 1	1	0.08	1.01	0.05	0.37	0.65
Pre 2 vs Post 2	1	0.10	1.10	0.06	0.37	0.97
Pre 3 vs Post 1	1	0.12	1.72	0.08	0.3	0.75
Pre 3 vs Post 2	1	0.08	1.02	0.06	0.37	0.49
Post 2 vs Post 1	1	0.01	0.16	0.009	0.95	0.72

Abbreviations: wks = weeks; d = days; v = versus; Df = degrees of freedom; SS = sum of squares; p-adj = adjusted p-value

Supplementary Table S5. The average relative abundance and standard errors of Orders and Families in the microbiome of young calves Pre 1 (2–3 days old), Pre 2 (5 weeks old), Pre 3 (12–13 weeks old, pre-weaning), Post 1 (12–13 weeks old, post-weaning), and Post 2 (13–14 weeks old).

Taxon	Age Group	Mean % RA	SE
Enterobacteriaceae	Pre 1	43.91	12.66
	Pre 2	0.40	0.19
	Pre 3	0.057	0.03
	Post 1	0.25	0.15
	Post 2	0.11	0.07
Enterobacterales	Pre 1	52.7	12.4
	Pre 2	0.8	0.2
	Pre 3	0.4	0.1
	Post 1	3.5	1.6
	Post 2	1.3	0.6
Pasteurellaceae	Pre 1	8.52	8.21
	Pre 2	0.10	0.04
	Pre 3	0.0004	0.0002
	Post 1	0.002	0.0009
	Post 2	0.0003	0.0002
Muribaculaceae	Pre 1	0.00007	0.00007
	Pre 2	7.91	1.73
	Pre 3	6.31	0.77
	Post 1	6.95	0.65
	Post 2	7.58	1.35
Bacteroidaceae	Pre 1	9.40	4.51
	Pre 2	15.14	1.37
	Pre 3	13.29	1.59
	Post 1	8.25	0.69
	Post 2	8.16	1.30
Butyricicoccaceae	Pre 1	10.62	3.79
	Pre 2	0.34	0.07
	Pre 3	0.65	0.09
	Post 1	0.56	0.05
	Post 2	0.56	0.06
Lachnospiraceae	Pre 1	5.99	2.078
	Pre 2	13.40	1.84
	Pre 3	12.81	0.69
	Post 1	11.62	0.41
	Post 2	11.78	1.20
Oscillospiraceae	Pre 1	0.42	0.24
	Pre 2	5.96	1.02
	Pre 3	12.90	1.11
	Post 1	12.50	0.85
	Post 2	12.73	0.69
Lactobacillaceae	Pre 1	6.66	4.50
	Pre 2	0.24	0.12
	Pre 3	0.01	0.002
	Post 1	0.003	0.002
	Post 2	0.002	0.001

Abbreviations: wks = weeks; d = days; v = versus; RA = relative abundance; SE = standard error

Supplementary Table S6. The average relative abundance and standard errors of the mechanisms in the resistome of young calves Pre 1 (2–3 days old), Pre 2 (5 weeks old), Pre 3 (12–13 weeks old, pre-weaning), Post 1 (12–13 weeks old, post-weaning), and Post 2 (13–14 weeks old).

Mechanism	Age Group	Mean % RA	SE
Tetracycline resistance ribosomal protection proteins	Pre 1	11.57	2.73
	Pre 2	36.66	3.10
	Pre 3	48.67	3.15
	Post 1	43.48	2.98
	Post 2	44.83	3.71
Tetracycline resistance MFS efflux pumps	Pre 1	4.61	0.21
	Pre 2	3.98	0.24
	Pre 3	3.78	0.39
	Post 1	2.25	0.15
	Post 2	2.04	0.42
23S rRNA methyltransferases	Pre 1	0.58	0.26
	Pre 2	4.29	0.45
	Pre 3	4.60	0.25
	Post 1	4.06	0.29
	Post 2	4.36	0.38
MLS resistance MFS efflux pumps	Pre 1	0.17	0.06
	Pre 2	1.42	0.16
	Pre 3	2.05	0.12
	Post 1	1.84	0.12
	Post 2	1.98	0.15
Multi-metal resistance protein	Pre 1	4.95	0.62
	Pre 2	1.75	0.41
	Pre 3	0.93	0.31
	Post 1	1.81	0.37
	Post 2	1.56	0.43
Multi-metal RND efflux pumps	Pre 1	2.80	0.15
	Pre 2	0.78	0.17
	Pre 3	0.42	0.13
	Post 1	0.76	0.16
	Post 2	0.63	0.18
Drug and biocide MFS efflux pumps	Pre 1	5.26	0.67
	Pre 2	2.24	0.54
	Pre 3	1.10	0.41
	Post 1	2.16	0.46
	Post 2	1.88	0.55
Drug and biocide RND efflux pumps	Pre 1	6.02	1.20
	Pre 2	2.13	0.46
	Pre 3	1.18	0.35
	Post 1	2.31	0.44
	Post 2	1.99	0.51

Abbreviations: wks = weeks; d = days; v = versus; RA = relative abundance; SE = standard error

Supplementary Table S7. Differential abundance analysis results using Analysis of Compositions of Microbiomes with Bias Correction (ANCOM-BC) with a Dunnet's type test. The microbial community results were completed at the taxonomic rank of Family, and the oldest group of calves (Post 2) was used as the reference.

	logFC				Standard Error				q-values				Sensitivity Testing Results			
Taxon	Pre 1	Pre 2	Pre 3	Post 1	Pre1	Pre2	Pre3	Post 1	Pre1	Pre2	Pre3	Post 1	Pre 1	Pre 2	Pre 3	Post1
Akkermansiaceae	-6.94	-3.13	-0.79	-0.59	1.1	1.27	0.7	0.71	1.71e-06	0.15	1.0	1.0	T	T	T	T
Atopobiaceae	-7.4	-1.35	-0.19	-0.16	0.75	0.68	0.49	0.45	5.02e-11	0.43	1.0	1.0	T	F	T	T
Bacillaceae	-3.91	-3.24	-2.29	0.2	0.98	0.81	0.71	0.79	0.002	0.002	0.02	1.0	T	T	T	T
Coriobacteriaceae	2.56	4.6	2.97	-0.03	1.5	1.13	0.82	0.87	0.71	0.003	0.01	1.0	T	T	F	T
Corynebacteriaceae	-0.8	0.39	1.2	1.86	0.9	0.79	0.55	0.52	1.0	1.0	0.26	0.01	T	T	T	T
Eggerthellaceae	-2.34	0.36	0.0	-0.26	0.67	0.52	0.41	0.35	0.011	1.0	1.0	1.0	T	T	T	T
Erysipelatoclostridiaceae	-0.64	-0.97	-0.42	-0.13	0.84	0.45	0.38	0.39	1.0	1.0	1.0	1.0	F	T	T	T
Lactobacillaceae	5.58	3.54	2.14	0.03	1.2	0.78	0.5	0.57	0.0003	0.0004	0.001	1.0	T	F	F	T
Planococcaceae	-5.57	-4.91	-3.36	-0.69	0.92	0.65	1.1	0.99	9.11e-06	1.87e-07	0.027	1.0	T	T	T	T
Prevotellaceae	-8.1	0.35	-0.47	-0.38	0.78	0.42	0.4	0.36	8.74e-12	1.0	1.0	1.0	T	T	T	T
Streptococcaceae	6.49	3.76	0.71	-0.32	0.97	1.0	0.63	0.65	1.37e-06	0.005	1.0	1.0	T	T	F	T
Sutterellaceae	-2.74	-0.26	-0.25	-0.09	0.76	0.54	0.45	0.44	0.0081	1.0	1.0	1.0	T	T	T	T
Oscillospiraceae	-4.85	-1.39	-0.39	-0.2	0.94	0.35	0.25	0.24	6.16e-05	0.002	0.86	1.0	T	T	F	F
Enterobacteriaceae	6.35	0.12	-0.84	0.25	0.88	0.95	0.71	0.79	6.62e-08	1.0	1.0	1.0	T	F	F	F

Abbreviations: T = True; F = False

Supplementary Table 8. Differential abundance analysis results using Analysis of Compositions of Microbiomes with Bias Correction (ANCOM-BC) with a Dunnet's type test. The antimicrobial resistance gene community results were completed at the mechanism level, and the oldest group of calves (Post 2) was used as the reference.

	logFC				Standard Error				q - values				Sensitivity Testing Results			
Taxon	Pre 1	Pre 2	Pre 3	Post 1	Pre 1	Pre 2	Pre 3	Post 1	Pre 1	Pre 2	Pre 3	Post 1	Pre 1	Pre 2	Pre 3	Post 1
16S rRNA methyltransferases	-1.24	-0.09	0.18	-0.08	0.36	0.35	0.36	0.34	0.02	1	1	1	T	T	T	T
23S rRNA methyltransferases	-3.19	-0.01	0.12	-0.12	0.60	0.36	0.34	0.33	0	1	1	1	T	T	T	T
Aminoglycoside O-phosphotransferases	-1.21	0.05	0.15	-0.07	0.29	0.30	0.28	0.28	0	1	1	1	T	T	T	T
Bleomycin resistance protein	-3.31	-1.06	0.51	-0.66	0.76	0.86	0.91	0.70	0	1	1	1	T	T	T	T
Class D betalactamases	-4.77	-0.98	-0.17	-0.22	0.70	0.64	0.45	0.46	0	1	1	1	T	T	T	T
Lincosamide nucleotidyltransferases	-2.02	-0.09	0.27	-0.12	0.47	0.33	0.33	0.32	0	1	1	1	T	T	T	T
Mercury resistance protein	1.99	1.21	0.74	0.74	0.48	0.47	0.45	0.48	0	0.17	1	1	T	F	T	T
MLS resistance ABC efflux pumps	-1.74	-0.37	0.19	-0.19	0.47	0.32	0.32	0.29	0.01	1	1	1	T	T	T	T
MLS resistance MFS efflux pumps	-3.64	-0.34	0.10	-0.13	0.64	0.35	0.34	0.32	0	1	1	1	T	T	T	T
Sulfonamide-resistant dihydropteroate synthases	-0.76	0.11	-0.03	-0.15	0.29	0.27	0.27	0.28	1	1	1	1	F	T	T	T
Tellurium resistance protein	2.71	0.92	0.51	-0.74	0.60	0.58	0.59	0.68	0	1	1	1	T	T	T	T
Tetracycline inactivation enzymes	-4.70	-0.12	0.17	-0.10	0.80	0.42	0.42	0.39	0	1	1	1	T	T	T	T
Tetracycline resistance ribosomal protection proteins	-2.45	-0.18	0.14	-0.08	0.55	0.31	0.31	0.30	0	1	1	1	T	T	T	T
16S rRNA methyltransferases	-1.24	-0.09	0.18	-0.08	0.36	0.35	0.36	0.34	0.02	1	1	1	T	T	T	T
23S rRNA methyltransferases	-3.19	-0.01	0.12	-0.12	0.60	0.36	0.34	0.33	0	1	1	1	T	T	T	T

Abbreviations: T = True; F = False
